# Scoping review: climate change-related anxiety and worry in children, adolescents, and young adults

**DOI:** 10.1007/s00787-026-03026-2

**Published:** 2026-07-03

**Authors:** Kristin Rodney-Wolf, Pauline Eisenschmidt, Julian Schmitz

**Affiliations:** 1 Department of Clinical Child and Adolescent Psychology and Psychotherapy, Wilhelm-Wundt-Institute of Psychology, Leipzig University, Leipzig, Germany; 2 Leipzig, Germany

**Keywords:** Climate change, Climate anxiety, Climate worry, Children, Adolescents, Young adults

## Abstract

Climate change is increasingly recognised as a significant threat to mental health, particularly among younger populations. Despite growing research attention, systematic syntheses of this emerging field still remain limited. This scoping review examined the prevalence of these emotional responses and their associations with mental health outcomes and related factors. Following the Preferred Reporting Items for Systematic Reviews and Meta-Analyses extension for Scoping Reviews (PRISMA-ScR) guidelines, we systematically searched APA PsycInfo, PubMed, and Web of Science for studies published before December 2025. Eligible studies reported quantitative data on climate anxiety, concern, distress, or worry in community samples of individuals under the age of 25. A total of 51 studies met inclusion criteria. Prevalence estimates for the four constructs varied widely, ranging from 18% to 90%, reflecting inconsistencies in definitions and measurement tools. The available evidence suggests that climate-related anxiety and worry may be positively associated with reduced mental wellbeing, as well as with symptoms of depression and generalised anxiety disorder. Age, gender, socioeconomic status, place of residence, education level, climate change knowledge and exposure, and participation in climate action emerged as potential influencing factors. Future research using standardised measures and longitudinal methods, particularly in younger children, is needed to clarify the direction of these relationships and distinguish between adaptive and maladaptive responses. As the climate crisis continues to unfold, addressing its psychological impacts, especially on vulnerable populations, must become a public health priority.

## Introduction

### The climate crisis

Climate change is widely recognised as one of the most serious global threats to the environment, public health, social equity, world peace, and economic stability in the 21st century [1–3]. Climate change refers to long-term shifts in climate patterns, identifiable through changes in the average or variability of climate indicators over several decades or more. While natural factors can contribute to such changes, the current climate change is primarily driven by human activity, particularly greenhouse gas emissions from unsustainable energy and land use, as well as consumption-based lifestyles, altering the atmosphere in ways that surpass natural variability [3–5].

The consequences of climate change are far-reaching and already observable across multiple domains of the Earth system. These include rising temperatures, ocean acidification and sea-level rise, natural disasters and extreme weather events (e.g., heatwaves, droughts, wildfires, floods, hurricanes), biodiversity loss and disruptions to ecosystems, potentially triggering food and water insecurity, elevated mortality, economic instability, displacement, and conflict [3, 6–8]. Given the severity of these consequences, the current change in climate is also referred to as a climate crisis [6].

### Effects of the climate crisis on child, adolescent, and young adult health

The climate crisis poses significant risks to both physical and mental health. Physical health consequences include dehydration and malnutrition from water and food insecurity, injury from extreme weather events, heat- and pollution-related respiratory and cardiovascular conditions, and spread of waterborne and vector-borne diseases leading to increased mortality [8–13].

Beyond physical health, the climate crisis also significantly affects mental health through multiple pathways, as conceptualised in Doherty and Clayton’s model of climate change-related psychological impacts [14]. These include psychological distress and potential trauma following direct exposure to climate change-related extreme weather events and natural catastrophes, psychosocial consequences of the crisis (e.g., displacement, migration, and climate-related conflict), and indirect impacts arising from awareness of the climate crisis and its anticipated future consequences, including anxiety, worry, grief, and despair [13–18].

Children and adolescents are particularly vulnerable to the impacts of the climate crisis. Physiologically, their developing bodies and immune systems make them more susceptible to environmental hazards such as heat, pollution, and climate-sensitive diseases [9, 19, 20]. Notably, over 90% of the global disease burden attributable to climate change affects individuals under the age of 15 [21, 22]. Psychologically, ongoing brain development during childhood and adolescence increases vulnerability to early-life stressors. Exposure to climate change-related events during these sensitive periods can disrupt neurological, psychological, and social development, leading to cognitive impairments, emotional and behavioural difficulties, mental health conditions such as post-traumatic stress disorder (PTSD), depression, and anxiety, and a heightened risk for mental health problems later in life [20, 23, 24]. Social determinants further compound these risks. Children and adolescents living in low-income or otherwise disadvantaged regions, especially those with limited access to food, clean water, healthcare, and stable housing, are disproportionately affected by climate-related health impacts due to existing social, economic, and structural inequities [19]. These populations are also more likely to experience displacement, poverty, and other stressors that increase psychological vulnerability. Moreover, children born in recent years are projected to face significantly more climate-related extreme events over their lifetime than previous generations, including more frequent heatwaves, droughts, floods, and wildfires [25, 26]. Such experiences can interfere with healthy development from the prenatal phase through adolescence and young adulthood, increasing the risk of long-term physical and mental health consequences [23, 27]. Despite this heightened vulnerability, there remains a lack of comprehensive research focussing specifically on the mental health impacts of climate change in children and adolescents [28].

### Climate change-related anxiety and worry among children, adolescents, and young adults

The growing recognition of the psychological consequences of climate change has led to increased attention on emotional responses such as fear, helplessness, grief, anger, and guilt, often referred to as eco-emotions, climate change emotions, or climate emotions [14, 27, 29–33]. These emotions are not only linked to personal exposure and vulnerability to environmental threats but also stem from the awareness that climate change is occurring and broader concerns about global and intergenerational justice and the future of the planet [29, 34, 35]. Among these climate change-associated emotions, climate anxiety, climate worry, climate concern, and climate distress have emerged as key constructs in both scientific literature and public discourse [29–31, 34, 36, 37].

Despite their frequent use, these terms are often inconsistently defined and conceptually overlapping, and vary in whether they emphasise emotional, cognitive, or functional aspects of responding to climate change [30, 31, 35, 36, 38]. For example, while some authors use *climate anxiety* and *eco-anxiety* interchangeably, others distinguish between them, with the former referring specifically to anxiety related to anthropogenic climate change and the latter to distress linked more broadly to the ecological crisis (“environmental doom” [24] including biodiversity loss, pollution, environmental degradation) [30, 39–42]. As these distinctions are applied inconsistently and the concepts overlap, given that most environmental problems are caused by or contribute to climate change, we use them synonymously in this paper under the term *climate anxiety.* Here, climate anxiety refers to future-oriented apprehension and chronic, potentially existential fear in response to anticipated dangerous and uncertain consequences of the climate and ecological crisis, marked by feelings of uncertainty, unpredictability, and uncontrollability [24, 27, 30, 36, 37, 39–44]. *Climate worry*, in contrast, is understood as a more cognitive and typically less intense response involving hard-to-control, repetitive negative thoughts about future climate impacts on society, nature, animals, and future generations, conceptualised as an attempt at mental problem-solving [30, 31, 37, 38, 45]. *Climate distress* refers to an aversive emotional, mental, or somatic state resulting from failed adaptation to climate change as a stressor [32]. It is often used as an umbrella term encompassing various negative emotional reactions to climate change from mild discomfort to chronic distress, including experiences not limited to anxiety or worry [32, 33, 38]. Similarly, *climate concern* broadly describes negative emotional responses such as ongoing worry, anxiety, and distress due to the awareness of climate change-associated threats, while also more strongly encompassing attitudes, beliefs, values, and behavioural intentions related to addressing climate and environmental problems [33, 40, 47, 48]. Due to their conceptual overlap, we refer to these constructs collectively as climate change-related anxiety and worry. While all of them relate primarily to emotional responses to the anticipated negative future impacts of climate change and thus are consequence-based prospective and threat-related emotions, other emotions like eco-grief or eco-guilt are more commonly linked to environmental changes that have already occurred in the past [27, 30, 31, 34, 44, 46, 47].

Given their heightened vulnerability to the effects of the climate crisis, research suggests that children, adolescents, and young adults are also at increased risk of experiencing elevated levels of climate change-related anxiety and worry. An international survey among over 10,000 young people aged 16–25 revealed widespread concern about climate change, with many reporting that it negatively affects their daily lives. The findings also showed feelings of betrayal toward government actions [29]. This suggests that children and adolescents may be particularly vulnerable to climate change-related anxiety and worry, not only due to their developmental sensitivity, but also because of their perceived lack of agency in climate decision-making.

While a moderate level of climate anxiety or worry is regarded to be a non-pathological and adaptive reaction to the threat of the climate crisis, potentially motivating functional coping, pro-environmental behaviour, and political engagement, high or persistent levels of anxiety and worry have been linked to depression, PTSD, somatic symptoms, and impaired psychosocial functioning and overall mental health [18, 29–31, 35–38, 48, 49].

Recent research investigated potential individual and contextual factors that influence how children and adolescents experience climate change-related anxiety and worry. Crandon et al., applying Bronfenbrenner’s Ecological Systems Theory [50], argue that these emotional responses are shaped by nested environments, from immediate settings like family and school (micro-/mesosystem) to broader cultural and societal influences (exo-/macrosystem) [28]. According to their narrative review, individual factors include personal vulnerability, values, and temperament. Contextual risk factors at the microsystem-level include insecure attachment to parents, peer problems, and family experience of climate stressors. Fear-based or poor-quality education on climate change in school and socioeconomic disparities (mesosystem) may increase anxiety and worry. In the exosystem, media reports highlighting disaster and destruction, governmental inaction, social divide and conflict can amplify fears and anxiety. On the macrosystem level, disrupted connections to nature, spiritual or cultural values due to destruction and displacement may exacerbate climate change-related distress [35, 38, 51].

### The current review

Despite increasing research interest in climate change-related anxiety and worry, there remains a lack of standardised definitions and validated instruments for their assessment. This methodological inconsistency has led to heterogeneous findings regarding the prevalence, mental health effects, and influencing factors of these constructs in children and adolescents [18, 43]. Given the projected intensification of climate change effects and the particular vulnerability of children and adolescents, due to their longer cumulative exposure, developmental sensitivity, and limited influence on climate policy, it is necessary to better understand the psychological impact of climate change on this age group. While the primary focus of this review is on children and adolescents, young adults are included to capture a broader developmental perspective and ensure a sufficiently comprehensive evidence base.

The current scoping review aims to address the identified research gap by providing a systematic overview of existing empirical research on climate change-related anxiety and worry in children, adolescents, and young adults. Specifically, it examines the prevalence of climate anxiety, concern, distress, and worry, associated mental health problems, and mitigating factors. By synthesising the current body of evidence, this review seeks to identify knowledge gaps and offer guidance for future research and interventions aimed at mitigating the psychological impact of climate change on younger populations.

The following research questions are investigated in this paper:

1. What is the prevalence of climate change-related anxiety and worry among children, adolescents, and young adults worldwide based on the current state of research?
2. Which mental health problems are associated with climate change-related anxiety and worry in this population?
3. Which individual, social, or contextual factors have been reported to be associated with climate change-related anxiety and worry among children, adolescents, and young adults?

## Methods

### Literature research

This review was conducted in accordance with the guidelines proposed by the Preferred Reporting Items for Systematic Reviews and Meta-Analyses extension for Scoping Reviews (PRISMA-ScR) [52]. The protocol for this scoping review was registered retrospectively on the Open Science Framework (DOI: https://doi.org/10.17605/OSF.IO/MSQ8G). The initial literature search was conducted in the APA PsycInfo, PubMed, and Web of Science databases between December 2023 and February 2024. In December 2025 an update of the literature search was performed in the same databases.

The following search string was used to identify studies that examined climate-change related anxiety or worry in children, adolescents and young adults: (((TI=(“eco”) OR TI=(“ecological”) OR TI=(“climate”) OR TI=(“climate change”)) AND (TI=(“anxi*”) OR TI=(“fear”) OR TI=(“worry”) OR TI=(“distress”)) AND (TI=(“child*”) OR TI=(“adolesc*”) OR TI=(“young”) OR TI=(“youth”) OR TI=(“teenager*”))) OR ((AB=(“eco”) OR AB=(“ecological”) OR AB=(“climate”) OR AB=(“climate change”)) AND (AB=(“anxi*”) OR AB=(“fear”) OR AB=(“worry”) OR AB=(“distress”)) AND (AB=(“child*”) OR AB=(“adolesc*”) OR AB=(“young”) OR AB=(“youth”) OR AB=(“teenager*”))) NOT TI=(“review”)).

In the initial literature search, the second author manually screened the identified studies multiple times based on title, abstract, and method. The first author confirmed the literature search and inclusion process. Eligible studies were then textually reviewed by the first and second author, and the results were summarised in tables. The update of the literature search was conducted by the first author and followed the same screening and review process.

Results were filtered further using the following inclusion criteria: The studies had to be peer-reviewed journal articles on the topic of climate-change related anxiety and worry in children, adolescents, and young adults written in the English language. They should present quantitative primary data of community samples of children, adolescents or young adults aged 0–24 years. The upper age limit of 24 years was defined in accordance with the United Nations’ definition of ‘childhood’ (< 15 years) and ‘youth’ (15–24 years) [53]. This range ensured adequate study inclusion, as preliminary searches using a narrower range (0–18 years) yielded an insufficient number of eligible publications. Studies investigating a broader age range were only included if results of the subpopulation up to the age of 24 was reported separately. Studies were only included if the full text was accessible or provided to the authors upon request. In order to obtain a global and comprehensive understanding of climate-change related anxiety and worry, no restrictions were imposed regarding time and location of data collection, and no temporal criterion was applied to the publication date.

Studies were excluded if they employed secondary research designs, such as meta-analyses or reviews. We also excluded studies with lower levels of evidence, including case reports, expert opinions, and commentaries. Intervention studies were only included if they reported data on the prevalence of climate change-related anxiety and worry, associated mental health outcomes, or other relevant factors, either at baseline or independently from any intervention or manipulation. Grey literature was not considered due to the absence of peer review. Additionally, studies that provided only qualitative data or focussed on clinical samples were excluded. The decision to focus exclusively on community samples was intentional, as this emerging field of research benefits from establishing prevalence and associated factors in the general population before addressing more specific clinical or potential high-risk groups.

**Fig. 1:**
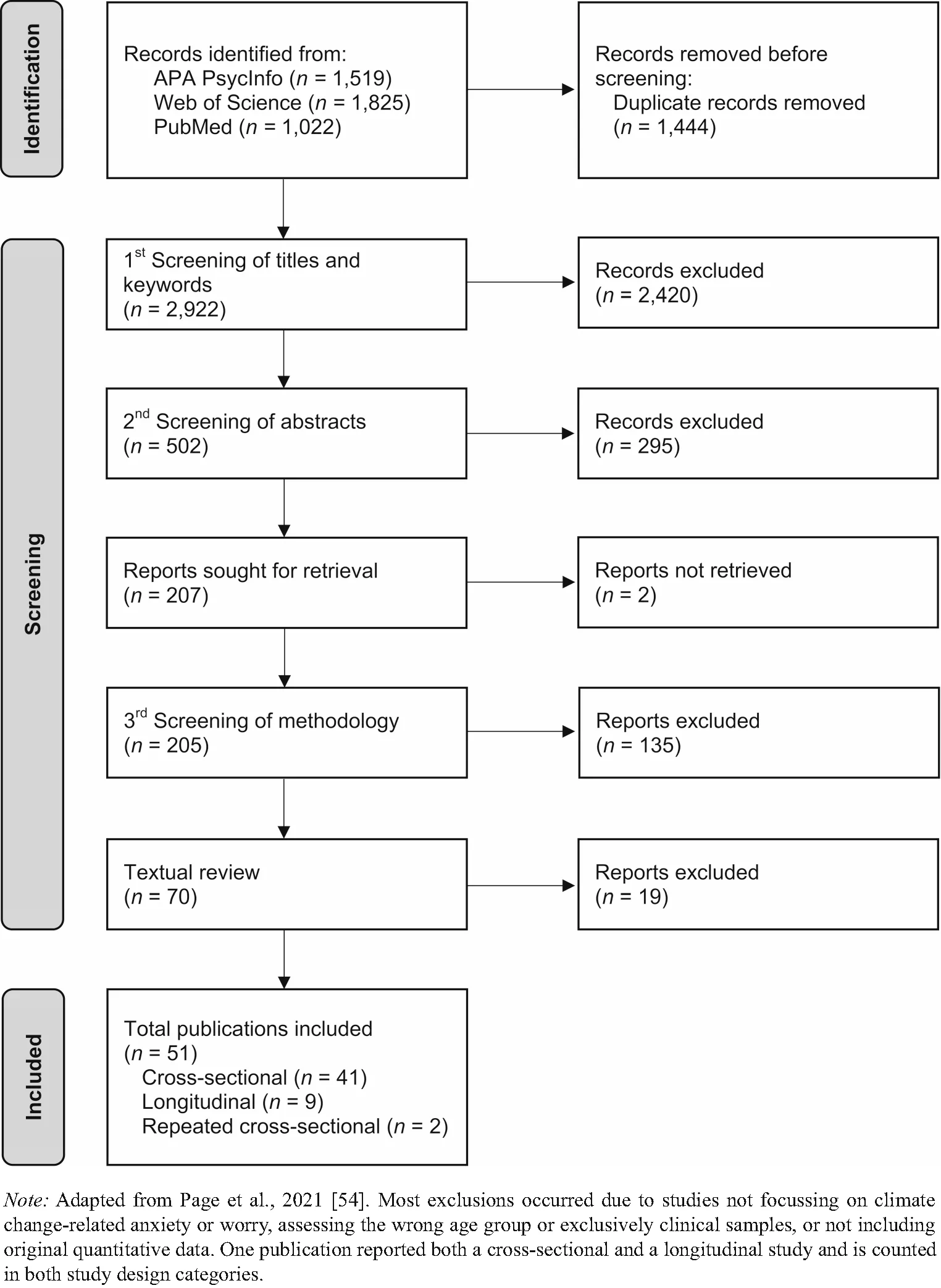
Process and Results of Literature Research (December 2023–February 2024 and December 2025)

The process of study selection is depicted in Fig. 1. 

### Synthesis of results

To synthesise the body of research regarding the first research question, we summarised findings on the prevalence and mean values of climate anxiety, concern, distress, and worry, including information on the age groups studied, assessment instruments used, and study locations. With respect to the second research question, we examined mental health problems which were identified to be positively associated with the four constructs and instruments used to assess them. To allow for more profound conclusions, we grouped the mental health outcomes into symptom categories as follows:

- General measures of mental health problems: psychological distress, low/negative wellbeing/life satisfaction/mental health.
- Internalising/emotional symptoms: symptoms of anxiety, symptoms of depression, symptoms of generalised anxiety disorder, symptoms of obsessive compulsive disorder, psychosomatic complaints (including symptoms of sleeping disorders), negative affect and emotions, negative expectations for the future.
- Externalising/behavioural symptoms: conduct problems, hyperactivity, peer problems, low prosocial behaviour, substance use.
- Self-harm and suicide-related symptoms: non-suicidal self-injury, suicidal thoughts, suicidal plans, suicidal attempts.

To answer the third research question, we collected factors that were found to influence or be associated with higher levels of climate change-related anxiety and worry. We included variables identified through a range of inferential statistical analyses, such as correlations, regressions, and group comparisons. Where studies found a negative association with climate change-related anxiety or worry (e.g., resilience), the inverse (e.g., lower resilience) is presented as a positively associated factor for consistency. We organised the associated factors into the following categories:

- Sociodemographic factors: e.g., gender, age, education, place of residence, household structure, socioeconomic status.
- Intraindividual factors: e.g., physical and mental health status, personality traits, cognitive factors, emotion regulation strategies, activities.
- Parental/family factors: e.g., parental education, parental mental health, parental climate-related emotions.
- Climate change-related factors: e.g., perception of climate change, exposure to climate change-related consequences, climate change-related attitudes and behaviour.

## Results

### Sample characteristics

A total of 51 studies, published between 2021 and 2025 and involving more than 251,000 children, adolescents, and young adults, were included in this review. Forty-one of these studies employed a cross-sectional design with a single assessment, nine used a longitudinal, within-subjects design, and two used a repeated cross-sectional, between-subjects design. Most studies were conducted between 2019 and April 2025, with only three longitudinal/repeated cross-sectional studies including data from earlier periods (2009–2018) [55–57].

The included studies present data from 29 countries across six continents. The majority (*n* = 22) were conducted in Europe, with four from the United Kingdom [58–61], four from Sweden [57, 62–64], three from Italy [65–67], two each from Germany [68, 69] and Spain [70, 71], and one each from Finland [72], Greece [73], Ireland [74], the Netherlands [75], Norway [76], Poland [77], and Slovenia [78]. Eleven studies originated in Asia, specifically three in China [79–81], two each in the Philippines [82, 83] and Turkey [84, 85], and one each in Bangladesh [86], India [87], Pakistan [88], and Qatar [64]. Eight studies were conducted and in North America, five in Canada [56, 89–92] and three in the United States of America [93–95], while three studies originated in South America [93–95], specifically Brazil [96, 97] and Colombia [75]. Five studies were conducted in Australia [55, 98–101]. The remaining four studies were conducted in Africa, namely Kenya [102], Madagascar [103], Tanzania [104], and Uganda [105].

Nearly all studies included adolescents aged between 15 and 19 years (*n* = 49). Twenty-five studies also included children and younger adolescents aged between 10 and 14 years, while 18 investigated young adults aged between 20 and 24 years. Three studies assessed children aged between six and nine years. No study assessed children under the age of six years.

In all studies, at least one of the four constructs, climate anxiety, climate concern, climate distress, and climate worry, was assessed. Climate anxiety was examined in 28 studies, climate worry in 21, climate distress in 10, and climate concern in five studies. They were most often assessed with self-report questionnaires (*n* = 50), using both single items (*n* = 15), self-developed or adapted (*n* = 12), and/or pre-existing multi-item instruments (*n* = 27). The latter included the Climate Change Anxiety Scale (CCAS) by Clayton and Karazsia [34], which was the most frequently used instrument for measuring climate anxiety in the included studies (*n* = 16). Other validated instruments used include the Hogg Eco-Anxiety Scale (HEAS) by Hogg et al. [106] (*n* = 5), the Climate Change Worry Scale (CCWS) by Stewart [45] (*n* = 4), and the Climate Distress Scale by Reser et al. [107] (*n* = 3).

### Overview of included studies

Table 1 provides a comprehensive overview of the 51 included studies.

**Table 1:** Overview of Included Studies

No.	Authors	Survey period	Country	*M*_Age_(*SD*_Age_)/ *Range*_Age_ in years	Study type	*N*	Constructs	Assessment of climate change-related anxiety or worry	Main findings
1.	Aruta, 2024 [82]	Not specified	Philippines	*M* = 19.19 (*SD* = 1.02) *Range* = ≥18	Cross-sectional	467 University students	Climate anxiety	Self-report Online questionnaire Climate Change Anxiety Scale (CCAS) by Clayton and Karazsia (2020), 13 items, five-point Likert scale (1[never]–5[almost always])	Mean climate anxiety score: M = 2.20 (SD = 0.77) (possible range = 1–5) Positive associations between climate anxiety and environmental concern and climate action
2.	Avan and Vural, 2025 [84]	03–06/2024	Turkey	*M* = 16.10 (*SD* = 1.13) *Range* = 14–17	Cross-sectional	833 Adolescents	Climate anxiety	Self-report Paper-pencil questionnaire CCAS, 10 items, five-point Likert scale (1[never]–5[almost always])	Positive associations between climate anxiety and depressive and insomnia symptoms
3.	Aylward et al. 2025 [92]	04/2022–03/2023	Canada	*Range* = 13–34 (only data for ages 13–24 reported here)	Cross-sectional	409 Adolescents (i.e., 13–18 years), young adults (i.e., 19–24 years)	Climate worry	Self-report Online questionnaire or telephone interview Adapted Climate Distress Scale (CDS^b^) by Searle and Gow (2010), 13 items on emotional responses to climate change (e.g., worried, anxious, depressed, concerned, stressed), five-point Likert scale (1[not at all]–5[extremely]), nine items loading on factor “climate change worry” and three on factor “climate change hopelessness”	Mean adolescent climate worry score: *M* = 2.70 [moderate], *CI* [2.43, 2.98] (possible range = 1–5) Mean young adult climate worry score: *M* = 2.69 [moderate], *CI* [2.39, 2.98] 63% of adolescents and 53% of young adults reported being at least moderately worried about climate change Effects in full sample (13–34 years): higher climate worry in non-binary than female and male participants, in female than male participants, in adolescents, during winter, in respondents with higher household income, living in urban areas, residing in Alberta or Yukon, reporting direct and/or indirect climate change exposure, and in respondents identifying as Indigenous and/or White
4.	Baker et al. 2021 [98]	T1: 11/2019 T2: 01–02/2020	Australia	*Range* = 11–17	Repeated cross-sectional	T1: 90 T2: 51 Parents and teachers	Climate anxiety Climate distress	Parent- and teacher-report Online questionnaire Single item, five-point Likert scale (1[very high]–5[very low])	Mean child/adolescent climate anxiety/distress score at T2: *M* = 3.24 [moderate] (*SD* = 1.0) (possible range = 1–5) No significant change in climate anxiety/distress from T1 and T2 2/3 of respondents indicated that children/adolescents were experiencing at least moderate distress/anxiety Sources for children’s and adolescents’ distress/anxiety: extreme weather events (32%), uncertainty about the future (29%), harm to animals (26%), lack of agency/trust in adults (25%), pollution/waste (14%), and harm to plants/deforestation (8%) Positive associations between climate anxiety/distress and higher age, desire and frequency of talking about climate change, and adult climate distress/anxiety
5.	Becht et al. 2024 [75]	Study 1: during COVID-19 pandemic Study 2: T1: summer 2022; T2: summer 2023 Study 3: summer 2023	Study 1–3: Netherlands Study 2 and 3: Colombia	Study 1: *M* = 13.85; *Range =* 12–17 Study 2 (T1): *M* = 14.09; *Range =* 12–17 Study 3 (T1): *M =* 17.68; *Range =* 16–18	Study 1 and 3: cross-sectional Study 2: longitudinal	Study 1: 259 Study 2: T1: 1,374; T2: 931 Study 3: 537 Adolescents	Climate anxiety	Self-report Online questionnaire Study 1 and 3: CCAS, 10 items, five-point Likert scale (1[never]–5[almost always]) Study 2 (T1) and 3: two items from the Achievement Emotions Adjective List by Romanello et al. (2022), five-point Likert scale (1[not at all]–5[extremely])	Study 1: mean climate anxiety score: *M* = 1.36 [low] (*SD* = 0.51) (possible range = 0–4) Study 2 (T1): mean climate anxiety score: *M* = 2.78 [moderate] (*SD* = 0.99) (possible range = 1–5) Study 3: mean climate anxiety score: *M* = 0.95 [low] (*SD* = 0.78) (possible range = 0–4)/2.59 [low] (*SD* = 1.08) (possible range = 1–5) [low] Study 1–3: positive association between climate anxiety and pro-environmental behaviour (public and private sphere), environmental efficacy, and symptoms of generalised anxiety disorder
6.	Błaszczak et al. 2025 [77]	Not specified	Poland	*M* = 22.85 (*SD* = 2.58) *Range* = 18–33 (only data for age 18 reported here)	Cross-sectional	170 Adolescents (i.e., 18 years; *N* = 66) and university students (*N* = 104)	Climate anxiety	Self-report Inventory of Climate Emotions (ICE) by Marczak et al. (2023), 32 items on eight emotions regarding climate change (e.g., anger, guilt, powerlessness, anxiety, sorrow), five-point Likert scale (1[definitely no]–5[definitely yes])	Mean adolescent climate anxiety score: *M* = 13.06 (possible range = 5–20)
7.	Comoutos et al. 2025 [73]	03–06/2024 (six months after extreme flood event)	Greece	*M* = 12.35 (*SD* = 1.38) *Range* = 9–16	Cross-sectional	1,021 Children and adolescents	Climate anxiety	Self-report Hogg Eco-Anxiety Scale (HEAS) by Hogg et al. (2021), 13 items, four-point Likert scale (0[not at all]–3[nearly every day])	Mean climate anxiety score: *M* = 0.98 [low] (*SD* = 0.60) (possible range = 0–3) Negative association between climate anxiety and wellbeing
8.	Donati et al. 2025 [65]	School year 2022–2023	Italy	*M* = 16.37 (*SD* = 1.25) *Range* = 13–21	Cross-sectional	1,846 High school students	Climate worry	Self-report Online questionnaire Climate Change Worry Scale (CCWS) by Stewart et al. (2021), 10 items, five-point Likert scale (1[never]–5[always])	Higher climate worry in female (*M* = 26.83 (*SD* = 8.58)) than male students (*M* = 24.43 (*SD* = 8.07)) and in older (*M* = 26.14 (*SD* = 8.62)) than younger students (*M* = 24.72 (*SD* = 7.97)) (possible range = 10–50)
9.	Ediz and Yanik, 2023 [85]	03–05/2023	Turkey	*Range* = 15–24	Cross-sectional	306 (103 climate activists, 203 non-activists) High school and university students	Climate anxiety	Self-report Online questionnaire CCAS, 13 items, five-point Likert scale (1[never]–5[almost always])	Mean climate anxiety score: *M* = 3.69 [moderate] (*SD* = 1.51) (possible range = 1–5) Higher climate anxiety in climate activists (*M* = 4.38 [high] (*SD* = 1.60)), than non-activists (*M* = 3.34 [moderate], (*SD* = 1.33)) Positive associations between climate anxiety and previous psychological support, knowledge about climate change, and participating in climate action Negative associations between climate anxiety and age and educational status (higher anxiety in 15–19-year-olds than 20–24-year-olds and in high school students than university students)
10.	Finnegan, 2023 [58]	2021	United Kingdom	*M* = 16.96 *Range* = 16–18	Cross-sectional	512 Secondary school students (12th and 13th grade)	Climate anxiety Climate distress Climate worry	Self-report Online questionnaire Scale on climate distress adapted from Wahlström et al. (2019, 2020) (negative emotions regarding climate change, distress as total score), seven items, five-point Likert scale (1[not at all]–5[very much]) Single item to assess feelings about the future, open question	Mean climate distress score: *M* = 3.62 [moderate] (*SD* = 0.96) (possible range = 1–5) 68% of participants reported being at least quite anxious and worried, 67% frustrated, 58% angry, and 57% fearful with respect to climate change 30% of participants reported feeling hopeful about the future, 15% scared, 14% excited, 13% worried, and 12% uncertain Higher climate distress in female and in white students than in students from ethnic minorities Positive associations between climate distress and participation in environmental protest and school sustainability actions
11.	Ge et al. 2025 [79]	T1: 04/2022 T2: 04/2023 T3: 04/2024	China	*Range* = 16–18	Longitudinal	426 (matched over T1–T3) Adolescents	Climate anxiety	Self-report Paper-pencil questionnaire CCAS, seven items, seven-point Likert scale (1[never]–7[always])	Mean climate anxiety score at T3: *M* = 0.36 (*SD* = 0.17) (possible range = 0–1) Significant increase in climate anxiety from T1 to T2 and T2 to T3 Higher climate anxiety in female than male adolescents Positive association between climate anxiety and awareness of climate change
12.	Glynn et al. 2025 [74]	11–12/2024	Ireland	*Range* = 15–16	Longitudinal(randomised controlled trial)	83 Female students (43 intervention group (IG), 40 control group (CG))	Climate anxiety	Self-report Online questionnaire HEAS, 13 items, four-point Likert scale (0[not at all]–3[nearly every day])	Mean climate anxiety score at baseline: *M*_IG_ = 7.74 (*SD*_IG_ *=* 6.49), *M*_CG_ = 6.67 (*SD*_CG_ *=* 5.07) (possible range = 0–36) Positive association between climate anxiety and climate capability; positive association between changes in climate anxiety and climate capability in from baseline to follow-up
13.	Goldberg et al. 2025 [86]	09–12/2023 (after flood season)	Bangladesh	*Range* = 15–18	Cross-sectional	1,200 Adolescents	Climate distress	Self-report Ten statements on climate distress, dichotomous scale	Over half of adolescents expressed agreement with nine of ten statements expressing climate distress Higher agreement to seven of the ten climate distress statements in participants from Barisal (higher flood exposure, lower socio-economic status, less adaptive infrastructure) than from Dhaka (capital city, less flood exposure, lower socio-economic status) 98% of participants from Barisal agreed that their feelings about climate change negatively affect their daily life, 97% that their family’s security will be threatened, 95% that people have failed to take care of the planet, 95% that the future is frightening, 93% that humanity is doomed, and 93% that things they value the most will be destroyed (vs. 67%, 68%, 92%, 76%, 87%, and 58% in Dhaka, respectively)
14.	González-Muñoz, 2024 [70]	2023	Spain	*Range* = 12–13, 15–16	Cross-sectional	1,050 Secondary school students (1st and 4th year of compulsory secondary education/7th and 10th grade in K-12 model)	Climate worry	Self-report Eight items on emotions towards climate change (e.g., hope, optimism, anger, fear, worry), 10-point Likert scale (1[not at all]–10[very much])	Mean climate worry score: *M =* 3.71 [strong] (*SD* = 1.29) (possible range = 1–5) Higher climate worry in eco-school students (curricular greening) than non-eco-school students Positive association between climate worry and use of social networks, traditional and online media, and specialised sources (i.e., lectures, workshops) to seek information on climate change, participation in climate movements, strikes, environmental groups, and schools’ voluntary activities
15.	Hadfield et al. 2025 [103]	03/2024	Madagascar (rural areas affected from multidimensional poverty)	*M* = 15.30 *Range* = 10–24	Cross-sectional	83 Children, adolescents, and young adults	Climate worry	Self-report Structured interview CCWS, 10 items, five-point Likert scale (1[never]– 5[always])	Mean climate worry score: *M =* 36.26 [high] (*SD* = 7.48) (possible range = 10–50)
16.	Hagquist, 2023 [62]	12/2020–03/2021	Sweden	T1: *Range* = 15–16 T2: *Range* = 16–17	Longitudinal	T1: 3,033 T2: 3,068 9th grade students/1st grade upper secondary school students	Climate worry	Self-report Online or paper-pencil questionnaire Single item, five-point Likert scale (1[never]–5[almost always]), only at T2	34% of participants reported being at least often worried about climate change at T2 Higher climate worry in female students Positive association between climate worry and parental education and psychosomatic complaints
17.	Hensler et al. 2025 [59]	02–11/2024	United Kingdom	*Range* = 8–12	Cross-sectional	772 Children	Climate worry	Self-report Online questionnaire Four items on global warming worry and knowledge adapted from Boyes et al. (2009), four-point Likert scale (1[not worried at all]–4[very worried])	42% of participants reported being at least quite worried about climate change Higher climate worry in participants from White backgrounds than from Asian/Asian British and Other ethnic groups and in adolescents than primary school-aged children
18.	Hindmarch et al. 2025 [93]	2021–2022	United States of America	*Range* = 12–17	Cross-sectional	2,154 Adolescents	Climate distress	Single item, dichotomous scale	38.1% of participants reported climate distress Higher climate distress in female, gender non-conforming, and White participants from higher-income families, with adverse childhood experiences Positive association between climate distress and psychological distress in the past month, suicide ideation in the past year, social media usage, community involvement, and civic engagement
19.	Innocenti et al. 2025 [66]	Study 1: 03–04/2024 Study 2: 01–04/2025	Italy	Study 1: *M* = 16.13; *Range* = 14–18 Study 2: *M* = 16.12; *Range* = 14–18	Cross-sectional	Study 1: 250 Study 2: 250 High school students	Climate anxiety Climate worry	Self-report Online questionnaire Study 1 and 2: CCAS, 13 items, two subscales: “cognitive-emotional impairment” (eight items), “functional impairment” (five items), five-point Likert scale (1[never]–5[almost always]); CCWS, 10 items, five-point Likert scale (1[never]– 5[always]) Study 2: HEAS, 13 items, four subscales: “affective symptoms” (four items), “rumination” (three items), “behavioural symptoms” (three items), “anxiety about personal impact” (three items) four-point Likert scale (0[not at all]–3[nearly every day])	Study 1: mean climate anxiety score: cognitive impairment: *M =* 11.57 (*SD* = 5.23) (possible range = 8–40); functional impairment: *M =* 6.66 (*SD* = 3.33) (possible range = 5–25); mean climate worry score: *M =* 21.35 (*SD* = 8.91) (possible range = 10–50) Study 2: mean climate anxiety score: *M =* 18.15 (*SD =* 6.92) (possible range = 13–65); affective symptoms: *M =* 2.00 (*SD =* 2.25) (possible range = 0–12); rumination:, *M =* 1.39 (*SD =* 1.62) (possible range = 0–9); behavioural symptoms *M =* 1.02 (*SD =* 1.77) (possible range = 0–9); anxiety about personal impact *M =* 1.80 (*SD =* 2.00) (possible range = 0–9); mean climate worry score: *M =* 20.12 (*SD =* 7.70) (possible range = 10–50) Study 1 and 2: positive association between climate anxiety and climate worry and pro-environmental attitudes (the latter only for certain climate anxiety subscales); positive association between climate worry and pro-environmental attitudes
20.	Jimenez-Vasquez et al. 2025 [71]	2023	Spain	*M* = 14.0 *Range* = 12–18	Cross-sectional	1,065 Adolescents	Climate anxiety	Self-report Online questionnaire CCAS, 13 items, two subscales: “cognitive-emotional impairment” (eight items), “functional impairment” (five items), five-point Likert scale (1[never]–5[almost always])	Mean climate anxiety score: *M =* 16.19 [low] (*SD =* 5.44) (possible range = 13–65) Higher climate anxiety in female adolescents, younger adolescents (12–14 years), and participants from families with lower socio-economic status Positive associations between climate anxiety and emotional problems, difficulties in emotion regulation, depressive, anxiety (panic disorder, separation anxiety, generalised anxiety disorder), and obsessive-compulsive disorder symptoms Negative associations between climate anxiety and resilience and health-related quality of life
21.	Jones and Lucas, 2023 [99]	10/2021–02/2022	Australia	*Range* = 15–19	Cross-sectional	1,943 Adolescents	Climate concern Climate worry	Self-report Online questionnaire Single item, six-point Likert scale (1[not concerned at all]–6[extremely concerned]) Assessment of climate worry not specified	88.6% of participants reported being at least somewhat concerned about climate change (68.8% very or extremely) 77.7% reported feeling worried about climate change, 69.3% powerless, and 56.5% frustrated Positive association between climate conversations with older people and feelings of betrayal, uncertainty, and worry
22.	König et al. 2025 [68]	12/2023	Germany	*Range* = 12–17	Cross-sectional	1,021 Adolescents	Climate distress	Self-report Online questionnaire or structured interview Climate Change Distress and Impairment Scale (CC-DIS) by Hepp et al. (2023), 23 items, two subscales: “climate change distress” (15 items), “climate change impairment” (eight items), five-point Likert scale (1[strongly disagree]–5[strongly agree])	Mean climate distress score: *M* = 3.33 [moderate] (*SD* = 0.78) (possible range = 1–5) Higher climate distress in female than male participants, in participants with high than low levels of education and high social status than low social status
23.	Lass-Hennemann et al. 2024 [69]	05–10/2022	Germany	*M* = 14.15 *Range* = 12–16	Cross-sectional	3,998 Students (7th and 9th grade)	Climate distress	Self-report Online or paper-pencil questionnaire Eight items on negative emotions regarding climate change based on Hickman et al. (2021), five-point Likert scale (0[not]–4[extremely])	Mean climate distress score: *M* = 1.51 (*SD* = 1.02) (possible range = 0–4) Mean climate anxiety score: *M* = 1.18 (*SD* = 1.18) (possible range = 0–4) Positive association between climate distress and symptoms of depression and generalised anxiety, and lower self-related quality of life
24.	Lawrance et al. 2022 [60]	05–06/2020	United Kingdom	*M* = 21.0 *Range* = 16–24	Cross-sectional	530 Adolescents and young adults	Climate distress Climate anxiety Climate concern	Self-report Online questionnaire Climate Distress Scale (CDS^a^) by Reser et al. (2012), eight items, four-point Likert scale (0[not at all]–3[very much]) List of 18 emotions based on the Yale Program on Climate Change Communication (YPCCC), four-point-Likert scale (1[not at all]–5[very much])	Mean climate distress score: *M* = 13.08 (*SD* = 6.60) (possible range = 0–32) 45.7% of participants reported being at least moderately anxious, 75.6% at least moderately concerned due to climate change 51.52% indicated worry about lack of action against climate change Higher climate distress in female participants Positive association between climate distress and sense of personal responsibility, guilt, concern, anger, concern, disgust, shame, disappointment, helplessness, sadness, being upset by media coverage, climate change-related agency, and symptoms of generalised anxiety
25.	Le and Kelly, 2024 [94]	2021	United States of America	*M* = 14.6 *Range* = 12–17	Cross-sectional	1,169 Adolescents	Climate anxiety	Self-report Telephone and web interview Single item, dichotomous scale	38.2% of participants experiencing reported climate anxiety Higher climate distress in participants living in urban than rural areas Positive association between climate anxiety and civic engagement
26.	Leonhardt et al. 2022 [76]	2021	Norway	*Range* = 13–19	Cross-sectional	128,484 Lower (8th − 10th grade) and upper secondary school (1st − 3rd grade) students	Climate worry	Self-report Online questionnaire Single item, five-point Likert scale (1[not at all]–4[very])	37.6% of participants reported being at least quite worried due to climate change Higher climate worry among female participants, older students (higher grade), those living in more central areas, and those with parents with higher education Positive associations between climate worry and depressive symptoms, participation in organised leisure activities, and cannabis use Negative associations between climate worry and subjective wellbeing, expectation of a happy life, alcohol intoxication, and spending more than one hour per day on social media
27.	Man et al. 2024 [89]	2021	Canada	*Range* = 11–19	Cross-sectional	1,029 Students (7th − 12th grade)	Climate anxiety	Self-report Single item, five-point Likert scale	Higher climate anxiety in participants assigned female at birth than assigned male at birth and in gender-diverse participants than in cisgender participants Positive association between climate anxiety and psychological distress
28.	Matijasevich et al. 2025 [96]	2022	Brazil	*M* = 18.0 *Range* = 18	Longitudinal (birth cohort study)	3,432 Adolescents	Climate anxiety	Self-report Structured interview HEAS, 13 items, four-point Likert scale (0[not at all]–3[nearly every day])	At least one third of participants reported experiencing climate anxiety symptoms for at least several days for at least 10/13 items Higher climate anxiety in female participants, in participants identifying as Black, with fair or poor health, very poor sleep quality, who smoked, currently did not study, respondents from low-income families, whose mothers had two or more previous births, lower education levels, or were non-White, and those whose mothers experienced depressive symptoms (post-partum and currently) Positive association between climate anxiety and external locus of control, mental health problems, emotional symptoms, and perceived stress Negative association between climate anxiety and self-esteem, emotional regulation capacities, and years of mother’s education
29.	Ndetei et al. 2024 [102]	05–06/2022	Kenya	*M* = 16.13 *Range* = 13–23	Cross-sectional	2,652 High school students (1st − 4th grade)	Climate anxiety Climate worry	Self-report Paper pencil questionnaire Self-developed scale, five items, two domains: climate-related worry (five-point Likert scale (0[not worried]–4[extremely worried])), four negative key emotions about climate change (e.g., anxiety) (dichotomous response options)	47.4% of participants reported being at least moderately worried about climate change 35.4% felt anxious about, 36.4% afraid of climate change Higher climate worry and fear among female participants and participants from rural than urban areas Positive associations between climate worry and general mental health problems, emotional symptoms, conduct problems, peer problems, suicide thoughts, plans, and attempts Negative association between climate worry and prosocial behaviour Positive associations between climate anxiety and general mental health problems, emotional symptoms, conduct problems, hyperactivity, peer problems, suicide thoughts, plans, attempts, hyperactivity and peer problems
30.	Patrick et al. 2023 [100]	08–11/2020	Australia	*M* = 52.71 *Range* = 18–>75 (only data for ages 18–24 reported here)	Cross-sectional	5,483 Thereof 281 young adults (18–24 years)	Climate anxiety	Self-report Online questionnaire CCAS, 11 items, five-point Likert scale (1[never]–5[almost always], cut-off = 3[sometimes])	23.53% of young adult participants reported being at least sometimes impacted by climate anxiety Higher climate anxiety in young adults than in all older age groups Age-independent effects: higher climate anxiety among female than male participants, participants with direct experience of climate change, and participants residing in more socio-economically disadvantaged areas
31.	Plohl et al. 2023 [78]	11/2022–03/2023	Slovenia	*M =* 21.57 *Range =* 18–24	Cross-sectional	442 Young adults	Climate worry Climate anxiety	Self-report Online questionnaire CCAS, 13 items, five-point Likert scale (1[never]–5[almost always]) CCWS, 10 items, five-point Likert scale (1[never]– 5[always])	Mean climate worry score: *M* = 2.68 (*SD* = 1.10) (possible range = 1–5) Positive associations between climate worry/climate anxiety and anxiety, stress, neuroticism, perceived threat of climate change, support for climate policies, and behavioural engagement Positive associations between climate worry and climate anxiety Negative associations between climate worry/climate anxiety and psychological resilience, and political conservatism Negative associations between climate worry and social wellbeing
32.	Prencipe et al. 2023 [104]	01–03/2021	Tanzania	*Range* = 18–23	Cross-sectional	2,053 Young adults	Climate distress	Self-report Structured interview Single item, four-point Likert scale (1[not at all]– 4[extremely])	18% of participants reported at least moderate climate distress Higher climate distress among female, more educated, more religious, and older participants, an among participants working in extreme temperatures Positive associations between climate distress and depressive symptoms, and climate change awareness
33.	Qin et al. 2024 [80]	Not specified 2022–2023 (estimated)	China	Not specified *Range* = 12–17 (estimated)	Cross-sectional	1,851 Middle (1st − 3rd grade) and high school (1st − 2nd grade) students	Climate anxiety	Self-report Paper pencil questionnaire CCAS, 13 items, five-point Likert scale (1[never]–5[almost always])	Mean middle school climate anxiety score: *M =* 1.57 (*SD =* 0.61) (possible range = 1–5) Mean high school climate anxiety score: *M =* 1.67 (*SD =* 0.65) (possible range = 1–5) Negative association between climate anxiety and pro-environmental behaviours, green self-efficacy, and future self-continuity
34.	Rikner Martinsson and Ojala, 2024 [63]	2019–2020	Sweden	*M =* 17.91 *Range* = 16–22	Cross-sectional	474 Senior high-school students	Climate worry	Self-report Online questionnaire Self-developed scale, five items, six-point Likert scale (1[not at all]–6[to a large extent])	Mean climate worry score in “solution-oriented coping” group (derived from cluster analysis; 43%, high on problem- and meaning-focussed coping): *M =* 4.80 (*SD =* 0.86) (possible range = 1–6) Mean climate worry score in “uninvolved coping” group (24%, low in all coping strategies): *M =* 4.01 (*SD =* 1.40) Mean climate worry score in “avoidant coping” group (33%, high on de-emphasising and meaning-focussed coping): *M =* 3.52 (*SD =* 1.12) Higher climate worry in solution-oriented group than in uninvolved and avoidant group Higher climate worry in uninvolved group than in avoidant group
35.	Rizeq and Ojala, 2025 [64]	Sweden: spring 2021–spring 2022 Qatar: 01–04/2022	Sweden Qatar	*Range* = 16–19	Cross-sectional	411 Secondary school students (*n* = 277 from Sweden; *n* = 134 from Qatar)	Climate worry	Self-report Online questionnaire Single item, six-point Likert scale (1[not at all]–6[to a large extent])	Mean climate worry score in Swedish sample: *M =* 3.76 (*SD =* 1.27) (possible range = 1–6) Mean climate worry score in Qatari sample: *M =* 3.81 (*SD =* 1.22) Positive association between climate worry and climate-friendly behaviours (both samples), negative affect (both samples), and climate hope (only Qatari sample) Negative association between climate worry and life satisfaction (only Qatari sample)
36.	Rizzini and Neumann, 2025 [97]	09–12/2024	Brazil	*Range* = 12–18	Cross-sectional	200 Public and private school students	Climate worry Climate anxiety	Self-report Structured interview Two items, one with a five-point Likert scale (1[strongly disagree]–5[strongly agree]), one with a single-response checklist with three options (anxiety, fear, insecurity; other (free text); no negative feelings)	90% of participants reported being worried about climate change, 3% reported not being worried 68.5% of participants reported experiencing anxiety, fear, or insecurity regarding climate change, another 11.5% reported feeling worry, anguish, sadness, anger, and revolt
37.	Romano et al. 2024 [67]	Not specified 2022 (estimated)	Italy	*M* = 16.09 *Range* = 13–19	Cross-sectional	480 High school students	Climate anxiety	Self-report Online questionnaire Climate Anxiety Scale by Wullenkord et al. (2021) based on CCAS, 12 items, seven-point Likert scale (1[strongly disagree]–7[strongly agree])	Mean climate worry score: *M =* 19.41 (*SD =* 10.50) (possible range = 12–84) Positive association between climate anxiety and seeking information via social media on pro-environmental content and participating in pro-environmental movements
38.	Sciberras and Fernando, 2022 [55]	T4: 2009– 2010 T5: 2011–2012 T7: 2015–2016 T8: 2017–2018	Australia	T4: *Range* = 10–11 T5: *Range* = 11–12 T7: *Range* = 16–17 T8: *Range* = 18–19	Longitudinal (data from T4, T5, T7, and T8 used for this study)	T1: 4,983 T8: 3,037 Children and adolescents	Climate worry	Self-report Online questionnaire T4 and T5: single item, four-point Likert scale (1[not at all worried]–4[very worried]) T7 and T8: single item, five-point Likert scale (1[not at all]–5[very often])	75.4% of participants reported at least moderate climate worry from T4 to T8 Six patterns of climate worry over late childhood/adolescence: 13% persistent high worry, 24.9% moderate worry, 24.3% increasing worry, 13.2% slightly decreasing worry, 24.6% persistent low worry or steeply decreasing worry Higher depressive symptoms at T8 in persistent and slightly decreasing worry group than other groups Higher engagement with news and politics at T8 in persistent and increasing worry group than other groups
39.	Simon et al. 2022 [83]	Not specified 2020–2021 (estimate)	Philippines	*M* = 19.18	Cross-sectional	452 Undergraduate students at a private university	Climate anxiety	Self-report Online questionnaire CCAS, 13 items, two subscales: “cognitive-emotional impairment” (eight items), “functional impairment” (four items), five-point Likert scale (1[never]–5[almost always])	Mean climate anxiety score: cognitive-emotional impairment: *M* = 18.67 (*SD* = 6.81) (possible range = 8–40); functional impairment: *M* = 9.85 (*SD* = 9.85) (possible range = 5–25) Cognitive-emotional, but not functional impairment mediates the positive influence of direct experience of climate change on behavioural engagement in climate mitigation
40.	Soomro et al. 2024 [88]	09–12/2022	Pakistan (flood affected province)	*Range* = 6–19	Cross-sectional	356 Parents (*n* = 142) and students (*n* = 214)	Climate anxiety	Self- and parent report Paper pencil questionnaire CCAS, 13 items, five-point Likert scale (1[strongly disagree]–7[strongly agree])	Positive association between climate anxiety and climate change experience, personal vulnerability, disaster risk for health, stress level, public health concern, and symptoms of depression and generalised anxiety disorder
41.	Swahn et al. 2025 [105]	07–11/2023	Uganda	*Range* = 18–24	Cross-sectional	249 Young women	Climate anxiety	Self-report CCAS, 13 items, five-point Likert scale (1[never]–5[almost always])	21% of participants reported moderate to severe levels of climate anxiety Higher climate anxiety in older participants (21–24 years), in participants with higher education, and participants not living in multigenerational households Positive association between climate anxiety and symptoms of generalised anxiety disorder
42.	Teo et al. 2024 [101]	04–08/2022	Australia	*M =* 16.2 *Range =* 15–19	Cross-sectional	18,076 Adolescents	Climate concern	Self-report Online or paper-pencil questionnaire Single item, five-point Likert scale (1[not at all concerned]–5[extremely concerned])	46.4% of participants were at least somewhat concerned about climate change (52.6% very or extremely) Higher climate concern among female and gender-diverse participants, 18–19-year-olds, non-Indigenous individuals, full-time students, those living in suburbs or major Australian cities, participants experiencing housing instability and/or financial difficulties, and those with self-reported mental health conditions Positive associations between climate concern and psychological distress and negative outlook on the future (stronger association gender-diverse and Indigenous participants, individuals living in outer-regional or remote areas, and those without a previously diagnosed mental health condition)
43.	Tian et al. 2023 [81]	T1: 11/2021 T2: 08/2022	China	*Range =* 12–14 (T1)	Longitudinal	T1: 723 T2: 458 Middle school students	Climate worry	Self- and parent-report Online questionnaire Scale adapted from Lawson et al. (2019), four items, eleven-point Likert scale (0[not at all]–10[very much])	Mean climate worry score at T1: *M* = 7.40 (*SD* = 1.95) (possible range = 0–10) Positive associations between climate worry at T1 and children-led environmental communication and conservation behaviour at T1, carbon neutrality knowledge at T1, and parental climate worry at T2
44.	Turcotte-Tremblay et al. 2024 [90]	03–05/2022	Canada	*Range ≈* 12–17	Cross-sectional	45,362 High school students (7th − 11th grade)	Climate anxiety	Self-report Online questionnaire Two items adapted from CCAS, five-point Likert scale (1[never]–5[almost always])	9% of participants reported that they had difficulty sleeping due to climate change anxiety at least sometimes, 6% having interference with ability to work due to climate change anxiety at least sometimes Higher climate anxiety–related impairment (i.e., sleep difficulties, interference with ability to work) in participants identifying as female or neither female nor male compared with male participants, and in participants from less affluent families Positive association between climate anxiety–related impairment and symptoms of generalised anxiety disorder and environmental engagement Negative association between climate anxiety–related impairment and school connectedness
45.	Veijonaho et al. 2024 [72]	T1: 10–12/2020 T2: 10–12/2021	Finland	T1: *Range =* 11–14 T2: *Range =* 12–15	Longitudinal	3,002 Elementary and middle school students	Climate distress	Eight items adapted from CCAS and scale by Veijonaho and Samela-Aro (2022) items, six-point Likert scale (1[not true at all]–5[completely true])	9–10% of participants at T1 and T2 in “overburdened” group (derived from latent profile analysis; high climate distress, high climate change denialism), 21–23% in “emotionally involved” group (high cognitive emotional impairment, lower behavioural impairment), 17–20% in “denialists” group (low climate distress, high climate change denialism), 49–51% in “normative-carefree” group (low climate distress, low climate change denialism) Profiles were relatively unstable from T1 to T2 Higher depressive symptoms in overburdened group than other groups Higher pro-environmental behaviour in emotionally involved group than other groups Lower life satisfaction in overburdened and emotionally involved group than other groups
46.	Vercammen et al. 2023 [61]	08–10/2020	United Kingdom	*M =* 21.01 *Range =* 16–24	Cross-sectional	539 Adolescents and young adults	Climate distress Climate worry	Self-report Online questionnaire CDS^a^, eight items, four-point Likert scale (0[not at all]–3[very much]) Scale on worry themes, eight items (climate change, personal finances, work/career, study/school, politics, relationships, economy, COVID-19), five-point Likert scale (1[never]–5[very often])	63.3% of participants were at least moderately distressed by climate change Higher climate distress among participants currently diagnosed and/or receiving treatment for a mental health disorder, and people with a higher socio-economic position Lower climate distress among male participants Positive association between climate distress and guilt/shame, frustration about inaction, lack of control/agency, worry about own future, perceived negative impacts of climate change, participation in climate activism, improvement of health and wellbeing from adopting eco-friendly practices, gaining a sense of purpose/identity through climate action, feeling inspired by others’ climate actions, being able to influence others’ behaviour through one’s own climate action, and worry about climate, economy, politics, and COVID-19 pandemic Negative association between climate distress and subjective experience of “better” local weather conditions Positive association between climate worry and subjective experiences of negative climate impacts, climate distress, and lower generalised anxiety symptoms
47.	Vercammen et al. 2025 [95]	11/2021–01/2022	United States of America	*M =* 20.4 *Range =* 16–24	Cross-sectional	2,834 Adolescents and young adults	Climate anxiety Climate distress Climate worry	Self-report Online questionnaire HEAS, 13 items, four-point Likert scale (0[not at all]–3[nearly every day]) CDS^a^, eight items, five-point Likert scale (0[not at all]–4[very much]) Scale on emotions related to climate change, 21 items, four-point Likert scale (0–3) Scale on worry themes, eight items (climate change, personal finances, work/career, study/school, politics, relationships, economy, COVID-19), five-point Likert scale (1[never]–5[very often])	Mean climate anxiety score: *M =* 13.1 [low] (*SD =* 10.1) (possible range = 0–39) Mean climate distress score: *M =* 21.6 [moderate] (*SD =* 7.6) (possible range = 8–40) 69.4% of participants reported being at least moderately distressed due to climate change > 70% of participants reported being at least sometimes worried about climate change “Concerned” and “anxious” among the six highest-rated climate-related emotions Positive association between climate anxiety and direct experience of climate-related events and symptoms of generalised anxiety disorder Positive association between climate distress and direct experience of climate-related events Positive association between climate worry and direct experience of climate-related events
48.	Vergunst et al. 2024 [56]	T1: 2013 T2: 2015 T3: 2021	Canada	T1: *Range =* 15 T2: *Range =* 17 T3: *Range =* 23	Longitudinal (birth cohort study)	1,325 Young adults	Climate worry	Self-report Single item, four-point Likert scale (0[not at all worried]–3[extremely worried]) (only at T3)	84.9% of participants reported being at least somewhat worried, 43.2% at least very worried about climate change Higher climate worry in female participants than male participants Positive association between climate worry at T3 and child cognitive ability, adolescent and current symptoms of anxiety at T1–T3, current symptoms of depression at T3, and current self-harm at T3 Negative association between climate worry and adolescent aggressive-oppositional symptoms at T1 and maternal delinquency
49.	Wu et al. 2023 [91]	Spring 2021	Canada	*M =* 16.3 *Range =* 15–18	Cross-sectional	2,306 Adolescents	Climate anxiety Climate concern	Self-report Online questionnaire CCAS-S adapted from CCAS, four items, five-point Likert scale (1[never]–5[almost always]) Scale adapted from Herman and Korhonen (2017), three items, five-point Likert scale (1[disagree a lot]–5[agree a lot])	48.7% of participants reported having experienced climate anxiety at least rarely 75.8% of participants reported being concerned about the consequences of climate change Positive association between climate anxiety and general anxiety, depression, and climate concern Negative association between climate anxiety and mental wellbeing
50.	Wullenkord and Ojala, 2023 [57]	T1: 2010 T2: 2019–2020	Sweden	T1: *M =* 17.17; *Range =* 16–20 T2: *M =* 17.91; *Range =* 16–22	Repeated cross-sectional	T1: 321 T2: 474 High school students	Climate worry	Self-report Paper pencil questionnaire Scale by Ojala (2012), five items, two factors: “micro worry” (worry about negative consequences for oneself or one’s friends or family) and “macro worry” (worry about negative consequences for future generations, animals, nature, people in poorer countries), six-point Likert scale (1[not at all]– 6[very much])	Mean micro worry score at T2: *M* = 3.58 (*SD* = 1.42) (possible range = 1–6) Mean macro worry score at T2: *M* = 4.58 (*SD* = 1.36) (possible range = 1–6) Significant increase in climate worry from T1 to T2 Positive association between climate worry general negative affect, lower life satisfaction and climate pessimism, problem-focussed coping, and behavioural engagement Positive association only between micro climate worry and emotion-focussed coping (distancing)
51.	Yatirajula et al. 2023 [87]	07/2021	India	*M* = 20.76 *Range* = 16–24	Cross-sectional	536 Adolescents and young adults living in urban slums	Climate concern	Self-report Online questionnaire Self-developed scale, eight items, three-point scale (1[disagree]–3[completely degree])	Mean climate concern score: *M* = 9.8 (*SD* = 5.34) (possible range = 0–24) 51.7% of participants reported to be less worried about climate change since the begin of the COVID-19 pandemic, 31.2% worried the same, 17.2% worried more

The studies are sorted alphabetically based on the first author’s last name. Only constructs, instruments, and findings relevant to the research questions are depicted.

### Prevalence of climate change-related anxiety and worry

Table 2 presents findings on the prevalence of climate change-related anxiety, concern, distress, and worry, based on data from 23 studies. Prevalence was assessed using a range of methods, including dichotomous and Likert-type scales, as well as measures of both frequency and intensity.

**Table 2:** Prevalence of Climate Change-Related Anxiety and Worry in Children, Adolescents, and Young Adults

	Number of studies	Prevalence	Instruments	Age range in years	Country	Survey year
Climate anxiety	9	21.00% ** [105]	CCAS	18–24	Uganda	2023
		23.53% *** [100]	CCAS	18–24	Australia	2020
		> 33.33% *** [96]	HEAS	18	Brazil	2022
		35.40% * [102]	Single item	13–23	Kenya	2022
		38.20% * [94]	Single item	12–17	United States of America	2021
		45.70% ** [60]	Single item	16–24	United Kingdom	2020
		48.70% *** [91]	CCAS-S	15–18	Canada	2021
		68.00% ** [58]	Single item	16–18	United Kingdom	2021
		68.50% * [97]	Single item	12–18	Brazil	2024
Climate concern	4	46.40% ** [101]	Single item	15–19	Australia	2022
		75.60% ** [60]	Single item	16–24	United Kingdom	2020
		75.80% **** [91]	Three items	15–18	Canada	2021
		88.60% ** [99]	Single item	15–19	Australia	2021/22
Climate distress	6	18.00% ** [104]	Single item	18–23	Tanzania	2021
		30.00–33.00% ***** [72]	CCAS	11–15	Finland	2020/21
		38.1% * [93]	Single item	12–17	United States of America	2021/22
		> 50.00% * [86]	Ten items	15–18	Bangladesh	2023
		63.30% ** [61]	CDS^a^	16–24	United Kingdom	2020
		69.40% ** [95]	CDS^a^	16–24	United States of America	2021/22
Climate worry	10	34.00% *** [62]	Single item	15–17	Sweden	2020/21
		37.60% ** [76]	Single item	13–19	Norway	2021
		42.00% ** [59]	Single item	8–12	United Kingdom	2024
		43.20% * [56]	Single item	23	Canada	2021
		47.40% ** [102]	Single item	13–23	Kenya	2022
		53.00% ** [92]	CDS^b^	19–24	Canada	2022/23
		63.00% ** [92]	CDS^b^	13–18	Canada	2022/23
		68.00% ** [58]	Single item	16–18	United Kingdom	2021
		> 70.00% *** [95]	Single item	16–24	United States of America	2021/22
		75.40% ***** [55]	Single item	10–12; 16–19	Australia	2017/18
		77.70% ** [99]	n.a.	15–19	Australia	2021/22
		90.00% **** [97]	Single item	12–18	Brazil	2024

The studies are sorted by construct and ascending prevalence. Prevalence is reported as the proportion of respondents experiencing each construct of climate change-related anxiety and worry. Prevalence was measured using: a dichotomous scale (*); a categorical intensity scale, with responses rated at least “moderately” or equivalent (**); a categorical frequency scale, with responses rated at least “sometimes” or equivalent, except for [91], which reports prevalence based on responses of “rarely” or more (***); a categorical agreement scale, with responses rated at least “agree a little” (****); and a latent profile analysis including groups with at least “moderate” worry or “high” climate distress (*****). *CCAS* = Climate Change Anxiety Scale; *CCAS-S* = Climate Change Anxiety Scale – Short Form; *CDS*^*a*^ = Climate Distress Scale by Reser (2012); *CDS*^*b*^ = Climate Distress Scale by Searle and Gow (2010); *HEAS =* Hogg Eco-Anxiety Scale.

Only five studies examined changes in the prevalence of climate anxiety and worry over time. Baker et al. [98] did not find a significant change in climate anxiety among children and adolescents over a three-month period. In contrast, Ge et al. [79] and Wullenkord and Ojala [57] reported a significant increases in climate anxiety and worry in adolescents over two- and ten-year periods, respectively. Sciberras and Fernando [55] conducted a latent profile analysis of children’s and adolescents’ climate worry across nine years, identifying multiple developmental patterns, with 24% of participants showing increasing levels of worry and 20% showing decreasing levels. Veijonaho et al. [72] used latent profile analysis over a two-year period and found climate worry and climate denial profiles among younger adolescents to be relatively unstable over time.

Table 3 summarises findings from 26 studies reporting mean values of climate change-related anxiety and worry among children, adolescents, and young adults. These results were derived from a variety of validated instruments, adapted or self-developed scales, and single-item measures. Scores were converted to a common 5-point scale (range = 1–5, with higher values indicating higher climate change-related anxiety and worry) to facilitate comparison. Mean scores for climate anxiety ranged from *M* = 1.25 to *M* = 3.69, for climate worry from *M* = 1.41 to *M* = 4.04, and for climate distress from *M* = 2.51 to *M* = 3.62. Only one study investigated the mean for climate concern, with *M* = 2.63.

**Table 3:** Mean Values of Climate Change-Related Anxiety and Worry in Children, Adolescents, and Young Adults

Construct label	Number of studies	*M* (*SD*)	Instruments	Age range in years	Country	Survey year
Climate anxiety	14	1.25 (0.42) [71]	CCAS	12–18	Spain	2023
		1.33 (0.67) [66]	CCAS (functional impairment subscale)	14–18	Italy	2024
		1.36 (0.51) [75]	CCAS	12–17	Netherlands	n.a.
		1.40 (0.53) [66]	HEAS	14–18	Italy	2025
		1.45 (0.65) [66]	CCAS (cognitive impairment subscale)	14–18	Italy	2024
		1.57 (0.61) [80]	CCAS	n.a., ~ 12–15	China	n.a.
		1.67 (0.65) [80]	CCAS	n.a., ~ 15–17	China	n.a.
		1.74 (0.56) [74]	HEAS	15–16	Ireland	2024
		1.86 (0.72) [74]	HEAS	15–16	Ireland	2024
		1.95 (0.78) [75]	CCAS	16–18	Netherlands and Colombia	2023
		1.97 (1.97) [83]	CCAS (functional impairment subscale)	n.a. (*M* = 19.18)	Philippines	n.a.
		2.18 (1.18) [69]	Eight items	12–16	Germany	2022
		2.20 (0.77) [82]	CCAS	≥ 18	Philippines	n.a.
		2.31 (0.80) [73]	HEAS	9–16	Greece	2024
		2.33 (0.85) [83]	CCAS (cognitive impairment subscale)	n.a. (*M* = 19.18)	Philippines	n.a.
		2.34 (1.04) [95]	HEAS	16–24	United States of America	2021/22
		2.44 (0.68) [79]	CCAS	16–18	China	2024
		2.59 (1.08) [75]	Two items	12–17	Netherlands and Colombia	2022
		2.61 [77]	ICE	18	Poland	n.a.
		2.76 (1.00) [98]	Single item	11–17	Australia	2019/20
		2.78 (0.99) [75]	Two items	12–17	Netherlands and Colombia	2022
		3.69 (1.51) [85]	CCAS	15–24	Turkey	2023
Climate concern	1	2.63 (0.89) [87]	Eight items	16–24	India	2021
Climate distress	6	2.51 (1.20) [69]	Eight items	12–16	Germany	2022
		2.64 (0.83) [60]	CDS^a^	16–24	United Kingdom	2020
		2.70 (0.95) [95]	CDS^a^	16–24	United States of America	2021/22
		3.24 (1.00) [98]	Single item	11–17	Australia	2019/20
		3.33 (0.78) [68]	CC-DIS	12–17	Germany	2023
		3.62 (0.96) [58]	Seven items	16–18	United Kingdom	2021
Climate worry	11	1.41 (0.58) [67]	CCAS	13–19	Italy	n.a.
		2.01 (0.77) [66]	CCWS	14–18	Italy	2025
		2.14 (0.89) [66]	CCWS	14–18	Italy	2024
		2.44 (0.81) (male subsample) [65]	CCWS	13–21	Italy	2022/23
		2.47 (0.80) (younger subsample) [65]	CCWS	13–21	Italy	2022/23
		2.61 (0.86) (older subsample) [65]	CCWS	13–21	Italy	2022/23
		2.68 (0.86) (female subsample) [65]	CCWS	13–21	Italy	2022/23
		2.68 (1.10) [78]	CCWS	18–24	Slovenia	2022/23
		2.69/2.70 [92]	CDS^b^	13–24	Canada	2022/23
		2.82 (0.90) (avoidant coping group) [63]	Five items	16–22	Sweden	2019/20
		3.06 (1.14) [57]	Five items (micro worry subscale)	16–22	Sweden	2019/20
		3.21 (1.02) [64]	Single item	16–19	Sweden	2021/22
		3.25 (0.98) [64]	Single item	16–19	Qatar	2022
		3.61 (1.12) (uninvolved coping group) [63]	Five items	16–22	Sweden	2019/20
		3.63 (0.75) [103]	CCWS	10–24	Madagascar	2024
		3.71 (1.29) [70]	Eight items	12–13; 15–16	Spain	2023
		3.86 (1.09) [57]	Five items (macro worry subscale)	16–22	Sweden	2019/20
		3.96 (0.78) [81]	Four items	12–14	China	2021/22
		4.04 (0.69) (solution-oriented coping group) [63]	Five items	16–22	Sweden	2019/20

The studies are sorted by construct and ascending mean value (possible range = 1–5, with higher values indicating higher climate change-related anxiety and worry). Where necessary, values and standard deviations were linearly transformed to a 5-point scale to improve comparability across studies. Values in brackets indicate clarifications such as values referring to specific subgroups or subscale scores. *CCAS* = Climate Change Anxiety Scale; *CC-DIS* = Climate Change Distress and Impairment Scale; *CDS*^a^ = Climate Distress Scale by Reser (2012); *HEAS* = Hogg Eco-Anxiety Scale; *ICE* = Inventory of Climate Emotions.

### Mental health problems associated with climate change-related anxiety and worry

Table 4 displays the mental health problems that were identified to be positively associated with climate change-related anxiety, concern, distress, and worry across 25 studies. Only three studies identified negative associations between climate worry and mental health problems. Leonhardt et al. [76] reported that climate worry was associated lower rates of alcohol intoxication. Vercammen et al. [61] found an association between climate worry and reduced symptoms of generalised anxiety disorder, while Vergunst et al. [56] reported a negative association between climate worry in young adulthood with aggressive-oppositional symptoms in adolescence.

**Table 4:** Mental Health Problems Positively Associated with Climate Change-Related Anxiety and Worry

Mental Health Problems	Number of studies	Instruments	Climate change-related anxiety and worry
*General measures of mental health problems* [96]	*15*	SDQ [96]	Climate anxiety [96]
Presence of a mental health condition [61, 101]	2	Single question [61, 101]	Climate concern [101], climate distress [61]
Psychological distress [78, 88, 89, 93, 96, 101]	6	CDS^c^ [64], DASS-21 [78, 96], K6 [89, 93, 101], not specified [88]	Climate anxiety [78, 88, 89], climate concern [101], climate distress [93], climate worry [64, 78]
Low/negative wellbeing, life satisfaction, quality of life [57, 64, 69, 71–73, 76, 78, 91]	9	Cantril ladder [76], KIDSCREEN-10 [69, 71], MHC-SF [78], SLSS [57, 64], SWEMWBS [91], SWLS [72], WHO-5 [73]	Climate anxiety [71–73], climate distress [69], climate worry [57, 64, 76, 78]
*Internalising/emotional symptoms* [71, 96, 102]	*23*	SDQ [71, 96, 102]	Climate anxiety [71, 96, 102], climate worry [102]
Symptoms of anxiety [56, 71, 78]	3	DASS-21 [78], MIA [56], RCADS-30 [71]	Climate anxiety [71, 78], climate worry [56, 78]
Symptoms of depression [55, 56, 69, 71, 72, 76, 84, 88, 91, 104]	10	CES-D10 [104], DEPS-10 [72], HSCL [76], K10 [55], MIA [56], PHQ-8 [91], PHQ-9 [69], QDIA-SR [84], RCADS-30 [71], not specified [88]	Climate anxiety [71, 72, 84, 88, 91], climate distress [69, 104], climate worry [55, 56, 76]
Symptoms of generalised anxiety disorder [60, 69, 71, 75, 88, 90–92, 105]	9	GAD-2 [91], GAD-7 [60, 75, 90, 95, 105], SCARED [69], RCADS-30 [71], not specified [88]	Climate anxiety [71, 88, 90, 91, 95, 105], climate distress [60, 69]
Symptoms of obsessive-compulsive disorder [71]	1	RCADS-30 [71]	Climate anxiety [71]
Negative expectations for the future [61, 76, 101]	3	Single item [76, 101], 8-item scale [61]	Climate concern [101], climate distress [61], climate worry [61, 76]
Psychosomatic complaints, symptoms of insomnia [62]	2	Bergen Insomnia Scale [84], PSP-Scale [62]	Climate anxiety [84], climate worry [62]
Negative affect [57, 64]	2	CDS^c^ [57, 64]	Climate worry [57, 64]
Feelings of guilt, responsibility, helplessness, sadness, shame [60, 61]	2	Adapted scale from YPCCC [60, 61], adapted scale from CASPE [60]	Climate distress [60, 61]
*Externalising/behavioural symptoms*	*2*		
Conduct problems [102]	1	SDQ [102]	Climate anxiety [102]
Hyperactivity [102]	1	SDQ [102]	Climate anxiety [102]
Peer problems [102]	1	SDQ [102]	Climate anxiety [102], climate worry [102]
Low prosocial behaviour [102]	1	SDQ [102]	Climate worry [102]
Cannabis use [76]	1	Two items [76]	Climate worry [76]
*Self-harm and suicide-related symptoms*	*3*		
Non-suicidal self-injury [56]	1	Single item [56]	Climate worry [56]
Suicide-related thoughts, plans, and attempts [93, 102]	2	PDSQ [102], two items [93]	Climate anxiety [102], climate distress [93], climate worry [102]

*CDS*^c^ = Child Depression Scale; *CES-D10* = Center for Epidemiologic Studies Depression Scale – 10-item version; *DASS*−*21* = Depression Anxiety Stress Scales – 21-item version; *DEPS*−*10* = Depression Scale – 10-item version; *GAD*−*2* = Generalised Anxiety Disorder – 2-item version; *GAD*−*7* = Generalised Anxiety Disorder – 7-item version; *HSCL* = Hopkins Symptom Checklist; *K10* = Kessler Psychological Distress *Scale* – 10-item version; *K6* = Kessler Psychological Distress Scale – 6-item version; *KIDSCREEN =* KIDSCREEN Questionnaire – Health-Related Quality of Life in Children and Adolescents; *MHC-SF* = Mental Health Continuum – Short Form; *MIA =* Mental Health and Social Inadaptation Assessment for Adolescents; *PDSQ* = Psychiatric Diagnostic Screening Questionnaire; *PHQ*-*8* = Patient Health Questionnaire – 8-item version; *PHQ*-*9* = Patient Health Questionnaire – 9-item version; *PSP*-*Scale* = Psychosomatic Problems Scale; *RCADS*-*30* = Revised Child Anxiety and Depression Scale; *SCARED* = Screen for Child Anxiety Related Emotional Disorders; *SDQ* = Strengths and Difficulties Questionnaire; *SLSS* = Students’ Life Satisfaction Scale; *SWLS* = Satisfaction With Life Scale; *SWEMWBS* = Short Warwick-Edinburgh Mental Wellbeing Scale; *QDIA-SR* = Quick Depression Inventory for Adolescents-Self-Report Form; *WHO-5* = WHO-5 Well-Being Index.

### Factors associated with climate change-related anxiety and worry

Table 5 presents sociodemographic, intraindividual, parental/family, and climate change-related factors which were reported to be positively associated with climate anxiety, concern, distress, and worry across 43 studies.

**Table 5:** Factors Associated with Higher Levels of Climate Change-Related Anxiety and Worry

Associated factors	Number of studies	Climate change-related anxiety and worry
*Sociodemographic factors*		
Gender		
Female [56, 58, 60–62, 65, 68, 71, 76, 79, 89, 90, 92, 93, 100–102, 104]	19	Climate anxiety [71, 79, 89, 90, 96, 100], climate concern [101], climate distress [58, 60, 61, 68, 93], climate worry [56, 62, 65, 76, 92, 102]
Gender-diverse [89, 90, 101], gender non-conforming [93], non-binary [61, 92]	6	Climate anxiety [89, 90], climate concern [101], climate distress [61, 93], climate worry [92]
Ethnicity		
Indigenous (Canada) [92]	1	Climate worry [92]
Non-Indigenous (Australia) [101]	1	Climate concern [101]
White (United Kingdom, United States of America, Canada) [58, 59, 92, 93]	4	Climate distress [58, 93], climate worry [59, 92]
Black (Brazil) [96]	1	Climate anxiety [96]
Non-White (maternal; Brazil) [96]	1	Climate anxiety [96]
Age		
Early adolescence (vs. childhood and late adolescence) [59, 71]	2	Climate anxiety [71], climate worry [59]
Late adolescence (vs. early adolescence and young adulthood) [65, 76, 85, 92, 98, 101]	6	Climate anxiety [85, 98], climate concern [101], climate distress [98], climate worry [65, 76, 92]
Young adulthood (vs. adolescence and later adulthood) [100, 104, 105]	3	Climate anxiety [100, 105], climate distress [104]
Education		
Full-time school attendance [101]	1	Climate concern [101]
No current school attendance [96]	1	Climate anxiety [96]
Low school connectedness [90]	1	Climate anxiety [90]
Lower education level [85, 96]	2	Climate anxiety [85, 96]
Higher education level [68, 104, 105]	3	Climate anxiety [105], climate distress [68, 104]
Attendance at “eco-school” with environmental education [70]	1	Climate worry [70]
Place of residence		
Major city (Australia) [101]	1	Climate concern [101]
Central/urban area [76, 92, 94]	3	Climate anxiety [94], climate worry [76, 92]
Suburban area [101]	1	Climate concern [101]
Rural area [102]	1	Climate worry [102]
Flood-prone region (Bangladesh) [86]	1	Climate distress [86]
Socioeconomically disadvantaged area [86, 100]	2	Climate anxiety [86, 100]
Family size and household structure		
Higher maternal parity (i.e., mothers with two or more previous births) [96]	1	Climate anxiety [96]
Non-multigenerational household [105]	1	Climate anxiety [105]
Socioeconomic status		
Lower socioeconomic position/household income, financial difficulties [71, 90, 96, 101]	4	Climate anxiety [71, 90, 96], climate concern [101]
Housing instability [101]	1	Climate concern [101]
Higher socioeconomic position/household income [61, 68, 92, 93]	4	Climate distress [61, 68, 93], climate worry [92]
*Intraindividual factors*		
Physical health		
Fair or Poor physical health [96]	1	Climate anxiety [96]
Very poor sleep quality [96]	1	Climate anxiety [96]
Current smoking [96]	1	Climate anxiety [96]
Mental health		
Adverse childhood experiences [93]	1	Climate distress [93]
Lower psychological resilience [71, 78]	2	Climate anxiety [71, 78], climate worry [78]
History of psychological support/treatment [61, 85]	2	Climate anxiety [85], climate distress [61]
Low self-esteem [96]	1	Climate anxiety [96]
Personality traits		
Neuroticism [78]	1	Climate anxiety [78], climate worry [78]
Cognitive factors		
Cognitive ability [56]	1	Climate anxiety [56]
External locus of control [96]	1	Climate anxiety [96]
Lower future self-continuity [80]	1	Climate anxiety [80]
Emotion regulation		
Problem-focussed coping [57, 63]	2	Climate worry [57, 63]
Emotion-focussed coping [57]	1	Climate worry [57]
Meaning-focussed coping [63]	1	Climate worry [63]
Difficulties in emotion regulation, lack of coping strategies [63, 71, 96]	3	Climate anxiety [63, 71, 96]
Activities		
Participation in organised leisure or school-based voluntary activities [70, 76]	2	Climate worry [70, 76]
Religious services attendance [104]	1	Climate distress [104]
Low daily social media use (< 1 h) [76]	1	Climate worry [76]
Social media use [93]	1	Climate distress [93]
Engagement with news and politics [55]	1	Climate worry [55]
Civic engagement, community involvement [93, 94]	2	Climate anxiety [94], climate distress [93]
Concern about other social crises (economy, politics, COVID-19 pandemic) [61]	1	Climate distress [61]
*Parental/family factors*		
Parental education		
Lower parental education [96]	1	Climate anxiety [96]
Higher parental education [62, 76]	2	Climate worry [62, 76]
Parental mental health		
Maternal depressive symptoms (post-partum and current) [96]	1	Climate anxiety [96]
Parental climate-related emotions		
Parent’s or teacher’s climate anxiety, worry and distress [81, 98]	2	Climate anxiety [98], climate distress [98], climate worry [81]
*Climate change-related factors*		
Perception of climate change		
Knowledge and awareness of climate change [74, 79, 81, 85, 104]	5	Climate anxiety [74, 79, 85], climate distress [104], climate worry [81]
Exposure to information on climate change (in social networks, traditional and online media, and specialised sources) [60, 67, 70]	3	Climate anxiety [67], climate distress [60], climate worry [70]
Perceived threat of climate change [78]	1	Climate anxiety [78], climate worry [78]
Perceived personal vulnerability to climate change* [88]	1	Climate anxiety [88]
Exposure to climate change		
Direct experience of climate change impacts [61, 83, 86, 88, 92, 95, 100, 104]	8	Climate anxiety [83, 88, 95, 100], climate distress [86, 95, 104], climate worry [61, 92, 95]
Perceived negative climate impacts (e.g., environmental degradation) [60, 67, 70]	1	Climate distress [61], climate worry [61]
Seasonal exposure (winter; Canada) [78]	1	Climate worry [92]
Perceived climate-related health risks* [88]	1	Climate anxiety [88]
Climate change-related attitudes		
Pro-environmental attitudes [66]	1	Climate anxiety [66], climate worry [66]
Climate agency, environmental efficacy [60, 75]	2	Climate anxiety [75], climate distress [60]
Lower green efficacy [80]	1	Climate anxiety [80]
Climate pessimism [57]	1	Climate worry [57]
Climate hope [64]	1	Climate worry [64]
Environmental concern [82]	1	Climate anxiety [82]
Concern about public health impacts of climate change* [88]	1	Climate anxiety [88]
Climate change-related behaviour		
Communication about climate change [81, 98], climate conversations with older people [99]	3	Climate anxiety [98], climate distress [98], climate worry [81, 99]
Pro-environmental behaviour [58, 61, 64, 72, 74, 75, 78, 81–83, 90]	11	Climate anxiety [72, 74, 75, 78, 82, 83, 90], climate distress [58, 61], climate worry [64, 78, 81]
Less pro-environmental behaviour [80]	1	Climate anxiety [80]
Participation in environmental protests, climate activism [58, 61, 67, 70, 85]	5	Climate anxiety [67, 85], climate distress [58, 61], climate worry [70]
Support for climate policies and system change [74, 78]	2	Climate anxiety [74, 78], climate worry [78]
Psychological benefits of climate action (e.g., sense of purpose, wellbeing, inspiration, influencing others) [61]	1	Climate distress [61]

For synthesis and clarity, conceptually similar constructs were grouped and, where necessary, reframed using consistent terminology. *The respective study provided limited information on construct definitions and operationalisation.

Five studies also reported correlations between these constructs: specifically, between climate anxiety and climate concern [91], climate concern and climate distress [60], climate distress and climate worry [61], and between climate anxiety and climate worry [66, 78].

## Discussion

Climate change is increasingly recognised as a major global crisis with wide-ranging environmental and societal implications. Children, adolescents, and young adults appear to be particularly affected by the physical and psychological impacts of climate change, including climate change-related anxiety and worry. This scoping review synthesises the current literature on the prevalence of climate anxiety, concern, distress, and worry in individuals up to the age of 24 as well as its associations with mental health problems and other factors.

The review was conducted according to PRISMA-ScR guidelines [52] and is based on studies identified through searches in PubMed, APA PsycInfo, and Web of Science. It includes 51 primarily cross-sectional studies published between 2021 and 2025, encompassing data from over 251,000 participants aged six to 24 years from 29 countries across six continents, with the largest number of studies conducted in Europe and Asia.

### Prevalence of climate change-related anxiety and worry

In addressing the first research question on the prevalence of climate change-related anxiety and worry among children, adolescents, and young adults, we identified 23 relevant studies, most conducted between 2020 and 2024. Four studies were conducted each in Australia and the United Kingdom, three each in Canada and the United States of America, two in Brazil, and the remaining seven in Kenya, Tanzania, Uganda, Bangladesh, Finland, Norway, and Sweden. Additionally, 26 studies reported mean values of climate change-related anxiety and worry, covering comparable time spans and geographic regions. On average, children, adolescents, and young adults were found to experience low to moderate levels of climate anxiety, worry, concern, and distress. Prevalence rates for the four constructs of climate change-related anxiety and worry varied widely across studies, with moderate or higher levels reported by 18.0% to 90.0% of participants. Specifically, nine studies on climate anxiety reported prevalence rates ranging from 21.0% to 68.5%. Four studies on climate concern found rates between 46.4% and 88.6%, comparable to the range of 34.0% to 90.0% reported for climate worry in ten studies. For climate distress, six studies reported rates between 18.0% and 69.4%, aligning with the span for climate anxiety. These prevalence estimates must be interpreted in light of substantial methodological variability, ranging from validated multi-item instruments to adapted scales and non-standardised single-item measures. Studies relying on single-item measures generally produced less comparable prevalence figures, likely contributing to the wide range of reported prevalence rates. In contrast, studies using established instruments yielded more consistent prevalence estimates: three studies using the Climate Change Anxiety Scale (CCAS) by Clayton and Karazsia [34] reported prevalence rates between 21.0% and 33.0%, while two studies using the Climate Distress Scale (CDS) by Reser et al. [107] reported rates between 63.3% and 69.4%. Regarding systematic effects of time and location of assessment, no consistent pattern emerged, except in the case of climate worry. Two studies from Scandinavian countries reported the lowest levels (34.0% to 37.6%), while one Brazilian and two Australian studies reported the highest (75.4% to 90.0%), suggesting possible regional effects. Interestingly, three African reported the lowest levels of climate anxiety (21.0% and 35.4%) and climate distress (18.00%), alongside moderate levels of climate worry (47.40%), compared to studies from other continents. However, the small number of studies and methodological variability limit the strength of these conclusions. Regarding temporal changes in prevalence, longitudinal research remains limited, with evidence of five studies suggesting a potential increase in climate anxiety and worry over longer periods of several years, but not over shorter spans of a few months. Age-related comparisons of prevalence rates and mean values of climate change-related anxiety and worry across studies do not reveal consistent patterns, due to wide age ranges examined in several studies and considerable heterogeneity within age groups. However, the available data tentatively suggests a trend of increasing climate anxiety and worry from childhood into late adolescence and young adulthood, with the highest levels typically observed in older adolescents and young adults.

In addition to methodological variability, heterogeneity in prevalence may also arise from individual differences in children’s, adolescents’, and young adults’ responses to the climate crisis, influenced by individual and contextual risk and protective factors, which are examined in detail in our third research question. According to disaster research, individuals differ in their vulnerability and response to large-scale stressors (e.g., stress-resistance, transient distress with healthy adaption, breakdown without recovery, post-traumatic growth), influenced by factors such as pre-existing mental health, coping resources, family and social support, and broader ecological conditions [108–110]. Although the predominantly cross-sectional evidence in this review precludes causal conclusions or identification of individual trajectories, the reviewed studies suggest that factors on multiple levels of proximal and distal influence, consistent with Bronfenbrenner’s Ecological Systems Theory [50], are linked to climate change-related anxiety and worry. These include individual characteristics (e.g., physical and mental health status, resilience, coping styles), family influences (e.g., parental education, mental health, and climate-related emotions), and broader contextual factors (e.g., direct exposure to climate change-related impacts, media coverage, sociocultural environment). The limited longitudinal evidence does not allow conclusions about how these factors contribute to changes in climate change-related anxiety and worry over time, however, the available data suggests diverse individual trajectories.

Our findings are mainly consistent with results from the international studies by Hickman et al. [29] and Ogunbode et al. [111] which assessed climate-related emotions across 10 and 32 countries, respectively. In Hickman et al.‘s study, approximately 60% of respondents reported being very or extremely worried or anxious about climate change. In the study by Ogunbode et al., 28% of participants reported being very or extremely anxious, and 46% reported being very or extremely worried. All of these figures fall within the prevalence ranges identified in the present review. Country-specific analyses in both studies revealed substantial differences levels of climate anxiety and worry, with higher prevalence observed in countries that are poorer, located in the Global South, and more directly affected by climate change. These findings support our review’s finding of higher prevalence of climate worry in Brazil and Australia compared to Northern European countries, but contradict the relatively low prevalence rates reported in three African studies. However, it is important to note that the present review includes only a few studies on the prevalence of climate change-related anxiety and worry from countries that have been identified as having some of the highest reported levels, such as the Philippines, India, and Brazil [29, 111], and from countries in the Global South (excluding Australia) in general, which limits the generalisability of our findings and may distort prevalence estimates. The findings of the identified longitudinal studies align with previous research on adult populations, which found a gradual increase in climate worry over extended periods [112], but partially diverge from evidence suggesting that climate concern is a substantially stable construct [113]. This cautiously suggests that climate change-related anxiety and worry in childhood, adolescence, and young adulthood may be less stable than in later life.

Taken together, our findings indicate that climate change-related anxiety and worry are moderately to highly prevalent among children, adolescents, and young adults, although prevalence levels vary considerably across studies. Further research, especially using standardised definitions, validated instruments, and longitudinal approaches, is needed to enhance conceptual clarity, determine more precise prevalence estimates, and examine changes over time. In addition, it is essential to conduct research across a broader range of geographic and cultural contexts to better understand how local factors, such as regional effects of climate change, socioeconomic conditions, and cultural attitudes, may influence the experience of climate change-associated emotions.

### Mental health problems associated with climate change-related anxiety and worry

Regarding the second research question on mental health problems associated with climate change-related anxiety and worry, we identified 25 studies reporting a positive association between various mental health outcomes and climate anxiety, concern, distress, or worry. Most evidence was found for associations between the climate change-related anxiety and worry constructs and psychological distress (*n* = 6), reduced wellbeing, life satisfaction, and quality of life (*n* = 9), as well as symptoms of depression (*n* = 10) and generalised anxiety disorder (*n* = 9). In line with these findings, negative expectations for the future (*n* = 3) and feelings of guilt, helplessness, sadness, or shame (*n* = 2) were also reported to be associated with climate change-related anxiety and worry. Given that both depression and generalised anxiety disorder are characterised by mental preoccupation with prospective misfortune and a general negative outlook on the future, it is unsurprising that they appear to be linked to anxiety and worry about the consequences of climate change. However, a key aspect of both conditions is that the subjective future outlook is typically unrealistically negatively distorted. In the context of the existential threat posed by climate change, one could argue that anticipating and fearing negative future outcomes is neither necessarily inappropriate, nor inherently maladaptive or pathological. It is important to note that these results reflect the associations between climate change-related anxiety and worry and mental health in the general population of children, adolescents, and young adults. Due to the exclusion of exclusively clinical samples, our review is limited in its ability to more broadly assess the association between mental health outcomes and prevalence of climate change-related anxiety and worry in children, adolescents, and young adults with mental disorders.

As the majority of studies identified were cross-sectional, the direction of the association remains unclear. Current research does not yet clarify whether individuals with higher levels of depression or generalised anxiety symptoms are more prone to develop climate-related anxiety and worry, whether heightened climate anxiety and worry increase vulnerability to broader anxiety or depressive symptoms, or whether these associations reflect conceptual overlap, such as shared features of persistent, future-oriented worry in the context of a large-scale and largely uncontrollable global threat. Potential mechanisms through which climate change-related anxiety and worry may contribute to poorer mental health include chronic rumination about anticipated future harm and feelings of helplessness or reduced perceived control, both core features of depression and generalised anxiety disorder, particularly among children and adolescents with limited capacity to influence systemic responses to climate change. Climate change may also be conceptualised as a chronic stressor, characterised by repeated exposure through media, education, and direct or indirect experiences of climate-related impacts, cumulatively straining existing coping resources. The climate crisis may further challenge fundamental assumptions about safety, predictability, and future life opportunities, potentially interfering with developmental tasks such as identity formation and future planning. Importantly, these processes are likely bidirectional: pre-existing mental health difficulties may heighten sensitivity to climate-related threats, while persistent climate change-related anxiety and worry may exacerbate underlying vulnerabilities. However, given the predominance of cross-sectional evidence, these mechanisms remain theoretical and require longitudinal investigation. Other mental health outcomes, such as other anxiety disorders, psychosomatic complaints, substance abuse, hyperactivity, conduct problems, or suicidality were also examined. However, the limited number of studies addressing each of these constructs does not allow for clear conclusions.

Our findings are in line with previous research linking climate change-related anxiety and worry to depression, anxiety, lower general mental health, and negative emotions such as sadness, anger, helplessness, guilt, and fear [18, 29, 48].

In conclusion, climate change-related anxiety and worry appear to be positively related to symptoms of depression and generalised anxiety disorder, as well as reduced mental wellbeing. However, additional research is necessary to clarify the direction of these associations and to explore the relationships with other mental health outcomes.

### Factors associated with climate change-related anxiety and worry

While investigating the third research question, focussing on individual, social, or contextual factors associated with climate change-related anxiety and worry, we identified 43 studies reporting on such factors.

Regarding sociodemographic factors, age and gender emerged as key influencing factors. A multitude of studies reported higher levels of climate change-related anxiety and worry in female (*n* = 19) and gender-diverse individuals (*n* = 6), while no study reported higher levels in male participants. The findings on age are more heterogeneous with the majority suggesting the highest levels of climate change-related anxiety and worry in older adolescents (*n* = 6). Prior research on adult populations [114, 115] and three of the included studies found that young adults tend to report higher levels of climate anxiety. Combined, these findings suggest that climate change-related anxiety and worry peak in late adolescence and early adulthood, corresponding to the results of cross-study comparison reported above. This may be due to a combination of greater exposure to the anticipated long-term effects of climate change compared to older adults, as well as increased exposure to news and media and a more advanced capacity to understand complex, long-term climate risks compared to younger adolescents and children. Nevertheless, given the limited number and methodological heterogeneity of available studies, firm conclusions regarding developmental trajectories cannot be drawn. Although a small number of studies suggest a developmental increase in climate change-related anxiety and worry, longitudinal evidence remains insufficient to substantiate this trend.

Other sociodemographic factors, including ethnicity, education level, place of residence, and socioeconomic status, were also found to be associated with climate change-related anxiety and worry. However, only a limited number of studies examined each of these factors, and some findings were contradictory. For example, associations between socioeconomic status and climate change-related anxiety and worry were mixed, with studies reporting elevated levels across both higher and lower socioeconomic groups. Similarly, findings regarding place of residence were inconsistent. While some studies reported higher levels of climate change-related anxiety and worry among individuals living in central or suburban areas of large cities, others found elevated levels in rural or socioeconomically disadvantaged regions. Evidence concerning education level was equally mixed, with studies suggesting that both lower and higher educational attainment may be associated with increased levels of climate change-related anxiety and worry. As most of these findings come from a limited number studies, they should be interpreted with caution. The inconsistencies may also reflect more complex interactions among influencing factors, for instance, the role of climate change education, which can be tied to both age and access to higher education, or the experience of climate impacts, which may vary depending on one’s place of residence and socioeconomic resources. This complexity could lead to context-dependent effects. For example, living in rural, disadvantaged areas with greater exposure to climate-related harm may heighten concern and distress, while higher socioeconomic status and urban residence may increase awareness and knowledge, also contributing to anxiety and worry.

Findings on intraindividual factors were similarly diverse. Interest in news and politics and engagement in certain activities, such as organised leisure time activities, social media use, and civic engagement, were reported to be related to climate change-related anxiety and worry. In addition, personality traits, cognitive ability, cognitive styles, and emotion regulation strategies appeared as potential influencing factors. Notably, the most consistent evidence pointed to elevated levels of climate anxiety among individuals with current or past physical and mental health issues (*n* = 8).

Considering social factors, evidence was scarce but provided preliminary evidence that parental education, mental health, and climate-related emotions might be associated with their children’s emotional response to climate change.

With regard to factors more directly related to the experience of climate change, our findings suggest that the perception of climate change, including awareness, knowledge, media consumption, and perceived threat, seems to be an important influencing factor (*n* = 10). Additionally, direct experiences of a broad range of climate change-related events, including natural disasters such as floods, droughts, wildfires, hurricanes, and extreme temperatures, appear to be linked to higher levels of related anxiety across countries, including Australia, Bangladesh, Canada, Pakistan, the Philippines, Tanzania, and the United States of America (*n* = 8). Findings on climate change-related attitudes were limited and heterogeneous. Overall, greater environmental concern and stronger pro-environmental attitudes were associated with higher levels of climate change-related anxiety and worry (*n* = 3). Evidence regarding the role of agency- and future-oriented attitudes was mixed. While some studies found that climate hope and perceived climate agency were linked to higher levels of anxiety and worry (*n* = 3), others reported higher levels among individuals reporting climate pessimism or lower self-efficacy (*n* = 2). As discussed earlier, climate change-related anxiety and worry are not necessarily maladaptive and often serve as motivators for action. A considerable number of studies found that individuals with higher levels of climate change-related anxiety and worry were more likely to engage in problem-focussed coping, such as discussing climate issues, adopting pro-environmental behaviours, participating in activism, and supporting climate policies (*n* = 20). These findings align with earlier research indicating a link between climate anxiety and climate action across different age groups [37, 114–116]. This suggests a dual nature of climate anxiety: while it seems to be associated with poorer mental health outcomes, such as depressive and anxiety symptoms, it might also foster constructive engagement with the climate crisis. This is supported by evidence that climate action may buffer the impact of climate anxiety on depressive symptoms, although this positive effect may diminish if individuals feel their efforts are ineffective [48]. Further research is needed to determine when climate anxiety is detrimental to mental health and when it serves as a beneficial motivator for collective action.

As with the findings on mental health, it is important to note that most studies reviewed here were cross-sectional, limiting our ability to draw causal conclusions. This further highlights the need for longitudinal research on the topic.

In conclusion, we found that a range of factors, such as age, gender, mental and physical health, perception and experience of climate impacts, and climate action, seem to be associated with climate change-related anxiety and worry. Further research is essential to clarify the directionality of these relationships and the interactions among them. Moreover, future work should explore how to harness the motivating potential of climate anxiety while mitigating its negative effects on mental health.

### Strengths and limitations of the current review

To our knowledge, our review is the first to simultaneously examine the prevalence, associated mental health problems, and influencing factors of climate anxiety, worry, concern, and distress. This comprehensive approach allows for comparisons across constructs and advances the field beyond studies focussed on single constructs. The global scope of our study, including of data from 29 countries across six continents, enhances the generalisability of the results. Another key strength lies in the age-specific focus. By focussing on children, adolescents, and young adults, this review addresses a significant gap in existing research, which has largely concentrated on adult populations. Given the particular vulnerability of younger individuals to both the mental health impacts of climate change and its long-term consequences, this focus is especially important.

However, several limitations must be acknowledged. There is a potential for selection bias, as study eligibility and inclusion were primarily assessed by a single researcher. Additionally, publication bias may have influenced the results, as studies with non-significant findings are often underrepresented in the literature. To ensure scientific quality, only peer-reviewed and published articles were included, potentially excluding relevant data from grey literature. Because we did not statistically assess the extent of publication or reporting bias, the impact of these biases on our findings cannot be estimated.

Another limitation concerns the timing and place of data collection in the included studies. The conclusions drawn in this review are most representative of Western, educated, industrialised, rich, and democratic societies (WEIRD-bias) as more than half of the studies were conducted in Western Europe, Australia, and North America. Given that people living in the Global South, in socioeconomically disadvantaged areas, small island nations, and Indigenous communities are among the most impacted by climate change [3, 117], their underrepresentation in current research is a serious limitation of this review. Furthermore, we found only three studies on climate change-related anxiety or worry in children under 10 years old and therefore cannot draw meaningful conclusions about this age group.

The wide variability in results may be attributed to conceptual and methodological inconsistencies, including the primary use of non-standardised single-item measures and heterogeneity in participant samples, study locations, and timing of assessment. The use of single-item measures raises concerns about the reliability and content validity of the findings and introduces a risk of detection bias and thus both under- and overestimation of prevalence rates and associations. Moreover, the majority of studies were cross-sectional, preventing the identification of causal or temporal relationships between the outcomes and influencing factors. While cross-sectional designs are suitable for addressing our first research question, the associations identified in response to the second and third questions offer only preliminary insights and should not be interpreted as causal. Together, these methodological limitations need to be considered when interpreting the validity and generalisability of the conclusions drawn from this review.

It is important to note that this review focussed on community samples, excluding studies that exclusively investigated clinical populations with diagnosed mental health disorders. While this approach was chosen to provide an overview of climate change-related anxiety and worry within the general youth population, it limits the generalisability of our findings to those children, adolescents, and young adults with mental health disorders. As a result, the prevalence of climate change-related anxiety and worry and their associations with mental health problems may be underestimated.

Additionally, we found some evidence of intercorrelations among the four constructs studied: climate anxiety, worry, concern, and distress. Due to the limited number of studies on certain constructs, in particular climate concern and climate distress, and the scarcity of research examining multiple constructs simultaneously, we were unable to clearly differentiate them conceptually. This is unsurprising, given the heterogeneous definitions, overlapping conceptualisations, and similarities in assessment instruments. These constructs appear to measure general fear, stress, negative thoughts, and negative affect related to the climate crisis, rather than representing distinct psychological phenomena. Standardised definitions, conceptual frameworks, and more comparative research are necessary to ensure accurate differentiation and consistent terminology.

Finally, due to the scoping approach of our review, we observed considerable heterogeneity in the assessed constructs, associated factors, and methods used across studies. This variability prevents a quantitative synthesis. Thus, our results are based on a qualitative summary of the findings. The partially limited number of studies investigating each construct, mental health outcome, and associated factor also restricts our ability to draw firm conclusions, leaving some research questions insufficiently answered.

### Implications for further research

The current study shows that the existing literature often makes only minimal distinctions between the constructs of climate change-related anxiety, worry, concern, and distress, with terms sometimes used interchangeably. Future research should place greater emphasis on clearly defining and differentiating these constructs. Additionally, the limited use of standardised scales reduces the comparability of findings across studies. To address this, future research should prioritise the use of standardised instruments and provide clear guidelines for their scoring and interpretation in order to improve consistency. As previously noted, longitudinal studies, particularly including younger children, are needed to examine causal relationships or developmental trajectories, particularly in relation to mental health outcomes and influencing factors, and provide insights into the dynamics of climate change-related anxiety and worry over time and across development.

There is a pressing need for further research into the mental health outcomes and other factors associated with climate change-related anxiety and worry, as many of the conclusions drawn in this review are based on a limited number of studies. Future research is also urgently needed in geographic areas most affected by climate change, as they are currently underrepresented in the research landscape. As climate change is a global multi-systemic crisis unproportionally affecting disadvantaged communities, cross-disciplinary cooperation, including partnerships with communities that are often excluded from research is necessary to better understand psychological responses to climate change and identify culturally sensitive solutions [76]. Future research should also prioritise investigating vulnerable groups, e.g., clinical samples with pre-existing mental health conditions, as current preliminary evidence from community samples suggests they may be at elevated risk for climate change-related anxiety and worry.

Additionally, future research should explore and clarify the conflicting findings identified in this review. For example, inconsistencies in the relationship between place of residence, socioeconomic status, and education level in relation to climate change-related anxiety and worry suggest a need for more focussed investigation into contextual and demographic factors. This will be essential for identifying and supporting particularly vulnerable groups. Finally, it appears promising to investigate the contexts in which climate change-related anxiety and worry are adaptive, such as when they motivate collective action or contribute to a sense of purpose in climate engagement, and the contexts in which these experiences may become maladaptive and detrimental to mental health.

### Practical implications

This review demonstrates that climate anxiety, worry, concern, and distress affect a substantial portion of children, adolescents, and young adults worldwide. While these emotions may be adaptive reactions to the global threat of the climate crisis, serving to motivate climate action, they also appear to be linked to mental health problems such as depression and generalised anxiety disorder. Therefore, the goal should be to support the adaptive and motivating aspects of these responses, while minimising negative effects on mental health and preventing the development of clinically significant symptoms.

On an individual level, this involves normalising these emotions as valid responses to a real threat, which may reduce dysfunctional reactions like fatalism, paralysis, or denial [118]. When dealing with climate change-related anxiety and worry, individuals often rely on problem-focussed coping strategies, such as pro-environmental behaviours and participation in collective climate action, which can buffer negative mental health outcomes [48, 119]. However, such strategies may be insufficient against a global crisis requiring systemic change. Additional coping methods may be needed, as anxiety can persist while the perceived threat of climate change remains [118]. While collective action is valuable, small-scale adaptation behaviours at the personal level (e.g., preparing one’s home for flood risks, learning gardening techniques) may be more helpful to reduce feelings of anxiety by enhancing agency and reduce perceived vulnerability, but their effectiveness, especially for those not fearing direct harm, and suitability for younger children remain unclear [118].

On a societal level, how climate change is discussed by families, educators, and media can shape young people’s emotional responses. These conversations should be informed, empathetic, and age-appropriate, fostering optimism, hope, agency, self-efficacy, and empowerment rather than fear, uncertainty, or feelings of betrayal [99, 120, 121]. Poor communication, particularly if invalidating or alarmist, can intensify distress [99]. Given the severe and growing threat climate change poses to both physical and mental health, mental health professionals must be equipped to address climate-related impacts through adequate training, cultural competence, and effective psychological interventions [14, 122]. Current expert opinions and reviews identified key elements of such interventions, such as building personal resilience, enhancing emotion management, encouraging meaning- and problem-focussed coping, fostering social connection, and strengthening relationships with nature [123–125]. However, most of the existing interventions still lack evaluation and adaptations for children and adolescents. Some strategies, such as problem-focussed coping or connection to nature, show mixed outcomes and may have different effects on climate change-related anxiety and worry and pro-environmental behaviour [124].

Ultimately, the root cause of these emotions, the lack of sufficient climate action, must be addressed structurally. The international study by Hickman et al. reported a widespread sense of betrayal and dissatisfaction with governmental responses among young people, alongside a perception that previous generations have failed to protect the planet [29]. This highlights the critical need for meaningful political action to reduce climate-related psychological distress. The World Health Organization similarly emphasises health as a central argument for climate action, calling for health, wellbeing, equity, and human rights to be placed at the centre of climate action. Their recommendations include building resilient health systems, investing in clean energy and sustainable infrastructure, protecting and restoring nature and biodiversity, transforming economic and financial systems, and integrating health and climate policies [117].

## Conclusion

This scoping review of 51 studies indicates that climate change-related anxiety and worry are highly prevalent among children, adolescents, and young adults. These experiences were consistently associated with symptoms of depression, generalised anxiety disorder, and lower mental wellbeing. Higher levels of climate-related anxiety and worry were more frequently observed among older adolescents, young adults, female and gender-diverse individuals, while associations with education level, socioeconomic status, and place of residence appear less consistent and more complex. Moreover, anxiety and worry related to climate change were commonly linked to greater knowledge and awareness of climate change, direct and indirect exposure to its consequences, and engagement in climate action.

Future research should employ standardised instruments, longitudinal designs, and include younger children to build on current findings. This is essential for clarifying the directionality of the relationship between climate change-related anxiety and worry, mental health problems, and associated factors. It will also help distinguish when such anxiety and worry are an adaptive, motivating response to the climate crisis and when they become maladaptive and harmful to mental health. In the face of this global climate and health crisis, prioritising the protection of children’s and adolescents’ mental health is not only a matter of public health, but also one of intergenerational justice.

## Data Availability

All data supporting the findings of this study are available within the paper.
